# Novel Diamide-Based Benzenesulfonamides as Selective Carbonic Anhydrase IX Inhibitors Endowed with Antitumor Activity: Synthesis, Biological Evaluation and In Silico Insights

**DOI:** 10.3390/ijms20102484

**Published:** 2019-05-20

**Authors:** Mohamed A. Abdelrahman, Wagdy M. Eldehna, Alessio Nocentini, Silvia Bua, Sara T. Al-Rashood, Ghada S. Hassan, Alessandro Bonardi, Abdulrahman A. Almehizia, Hamad M. Alkahtani, Amal Alharbi, Paola Gratteri, Claudiu T. Supuran

**Affiliations:** 1 Department of Pharmaceutical Chemistry, Faculty of Pharmacy, Egyptian Russian University, Badr City, Cairo 11829, Egypt; mohamed.ashraf.eru@gmail.com; 2 Department of Pharmaceutical Chemistry, Faculty of Pharmacy, Kafrelsheikh University, Kafrelsheikh 33516, Egypt; 3 Department of NEUROFARBA, Section of Pharmaceutical and Nutraceutical Sciences, University of Florence, Polo Scientifico, Via U. Schiff 6, Sesto Fiorentino, 50019 Firenze, Italy; alessio.nocentini@unifi.it (A.N.); silvia.bua@unifi.it (S.B.); alessandro.bonardi@unifi.it (A.B.); 4 Department of NEUROFARBA, Section of Pharmaceutical and Nutraceutical Sciences, Laboratory of Molecular Modeling Cheminformatics & QSAR, University of Florence, Polo Scientifico, Via U. Schiff 6, Sesto Fiorentino, 50019 Firenze, Italy; paola.gratteri@unifi.it; 5 Department of Pharmaceutical Chemistry, College of Pharmacy, King Saud University, P.O. Box 2457, Riyadh 11451, Saudi Arabia; salrashood@ksu.edu.sa (S.T.A.-R.); mehizia@ksu.edu.sa (A.A.A.); haalkahtani@ksu.edu.sa (H.M.A.); amal.harbi@gmail.com (A.A.); 6 Department of Medicinal Chemistry, Faculty of Pharmacy, Mansoura University, Mansoura 35516, Egypt; ghadak25@yahoo.com

**Keywords:** anticancer activity, diamide-based benzenesulfonamides, molecular docking, selective hCAIX inhibitors, synthesis

## Abstract

In this work, we present the synthesis and biological evaluation of novel series of diamide-based benzenesulfonamides 5a–h as inhibitors of the metalloenzyme carbonic anhydrase (CA, EC 4.2.1.1) isoforms hCA I, II, IX and XII. The target tumor-associated isoforms hCA IX and XII were undeniably the most affected ones (*K*_I_s: 8.3–123.3 and 9.8–134.5 nM, respectively). Notably, diamides 5a and 5h stood out as a single-digit nanomolar hCA IX inhibitors (*K*_I_s = 8.8 and 8.3 nM). The SAR outcomes highlighted that bioisosteric replacement of the benzylidene moiety, compounds 5a–g, with the hetero 2-furylidene moiety, compound 5h, achieved the best IX/I and IX/II selectivity herein reported with SIs of 985 and 13.8, respectively. Molecular docking simulations of the prepared diamides within CA IX active site revealed the ability of 5h to establish an additional H-bond between the heterocyclic oxygen and HE/Gln67. Moreover, benzenesulfonamides 5a, 5b and 5h were evaluated for their antitumor activity against renal cancer UO-31 cell line. Compound 5h was the most potent derivative with about 1.5-fold more enhanced activity (IC_50_ = 4.89 ± 0.22 μM) than the reference drug Staurosporine (IC_50_ = 7.25 ± 0.43 μM). Moreover, 5a and 5h were able to induce apoptosis in UO-31 cells as evidenced by the significant increase in the percent of annexinV-FITC positive apoptotic cells by 22.5- and 26.5-folds, respectively.

## 1. Introduction 

Carbonic anhydrases (CA) are zinc metalloenzymes that play a pivotal role in most living organisms catalyzing the interconversion of carbon dioxide and water to bicarbonate and protons [1]. Seven distinct CA families (α-, β-, γ-, δ-, ζ-, η- and θ-CAs) are known to date [1,2]. Human encode 15 different α-CA isoforms among which some are cytosolic (CA I, CA II, CA III, CA VII and CA XIII), others are membrane bound (CA IV, CA IX, CA XII, CA XIV and CA XV), two of them are mitochondrial (CA VA and CA VB), and one isozyme is secreted in saliva (CA VI) [2]. They are involved in numerous physiological and pathological processes such as gluconeogenesis, lipogenesis, ureagenesis and tumorigenicity [2]. While inhibitors targeting hCA I are useful in retinal and cerebral edema, inhibitors targeting hCA II are used as diuretics, in the management of edema, as antiglaucoma agents, antiepileptic drugs, and also for the treatment of altitude sickness [1,2]. 

The hCA IX expression is usually induced by hypoxia in certain types of solid tumors, such as glioma, breast cancer and colon carcinoma [3,4]. Besides, inhibition of hCA IX was strongly associated with remarkable suppression of the growth of both primary tumor stages as well as metastases which makes the enzyme a validated target for the treatment of diverse cancers [5]. Accordingly, discovery of isoform hCA IX selective inhibitors stands out as a crucial step to establish a promising cancer therapy devoid of the classical adverse effects owing to isoforms hCA I and II inhibition.

**Figure 1:**
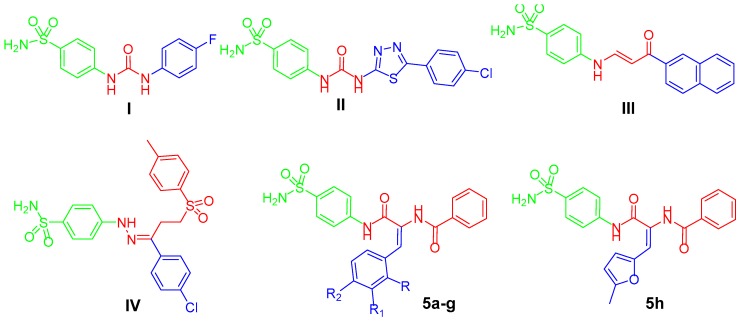
Structures of some reported benzenesulfonamides-based CAIs (**I**–**IV**) and the target compounds (5**a**–**g** and 5**h**).

While different CA inhibition mechanisms are known, the zinc binder sulfonamides undoubtedly represent the most important class, with many derivatives in clinical use for decades or clinical development in the last period, such as SLC-0111 (Figure 1) [6]. The CA inhibition mechanism with sulfonamides is mainly mediated by coordination of its deprotonated form (SO_2_NH^–^) to the positively charged Zn(II) ion from the enzyme active site. Moreover, the sulfonamide moiety engages two H-bonds: the NH^–^ group acts as donor, while the S=O as acceptor with T199 OG1 atom and backbone NH respectively. These binding features are common among the active site architectures for all the α-class fifteen human isozymes. 

Several approaches have been adopted for obtaining isoform-selective CAIs, with the “tail approach” being the most successful and exploited one (Figure 1) [7,8,9]. It consists in appending ‘‘tails’’, with a diverse chemical nature, to the aromatic/heterocyclic ring possessing the zinc binding sulfonamide group, in order to interact with amino acid residues from the middle and the rim parts of the active site cavity. In this context, several sulfonamide-based derivatives were successfully developed as isoform hCA IX selective inhibitors through utilization of “tail approach” (compounds Figure 1II–IV) [7,8,9]. 

Pursuing on our effort towards development of selective hCA IX inhibitors [10,11,12,13], herein we present the synthesis, and biological evaluation of novel series of diamide-based benzenesulfonamides 5a–h. The herein reported diamides possess a zinc anchoring moiety of the benzenesulfonamide type that was linked to benzylidene tails incorporating halogen, methyl or methoxy substituents to ensure suited SAR exploration regarding the hydrophobic region of the binding cleft. Also, the bioisosteric replacement approach was adopted to replace the benzylidene tails (compounds 5a–g) with the hetero 2-furylidene one (compound 5h, Figure 1). The amide functionality of the appended benzamide moiety was expected to engage contact with the amino acids residues of the “hydrophilic” region of the active site.

All the newly-synthesized diamides 5a–h were characterized and biologically tested against a panel of hCA I, II, IX and XII isoforms, using stopped-flow CO_2_ hydrase assay. Moreover, diamides 5a, 5b and 5h were evaluated for their anti-proliferative activity against renal cancer UO-31 cell line. Compound 5h was further investigated for its apoptosis induction potential in UO-31 cells. Furthermore, molecular docking investigations were carried out to provide insights for the binding interactions of the herein reported benzenesulfonamides within hCA isozymes II and IX.

## 2. Results and Discussion 

### 2.1. Chemistry

**Scheme 1:**
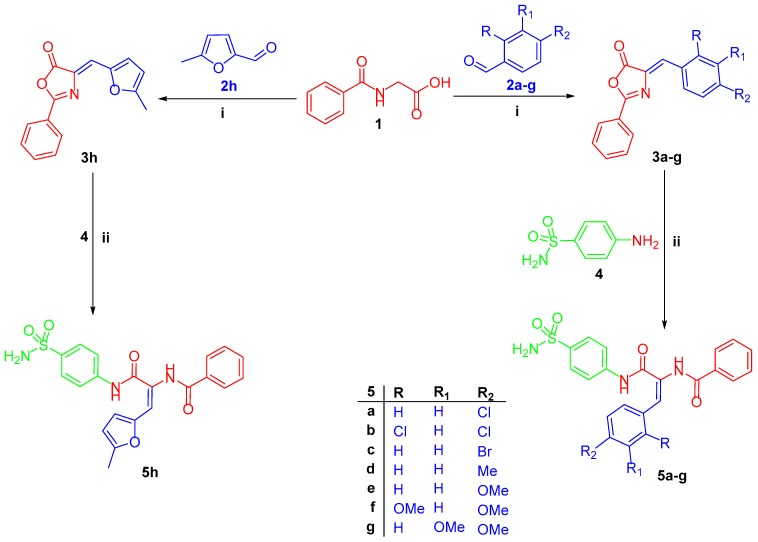
Synthesis of diamide-based benzenesulfonamides 5**a**–**h**; Reagents and conditions: (**i**) Acetic anhydride/Hünig’s base/reflux 3 hrs., (**ii**) Glacial acetic acid/CH_3_COONa/reflux 4 h.

The synthetic pathway adopted for the preparation of the target diamide-based benzenesulfonamides 5a–h was depicted in Scheme 1. The azlactone intermediates 3a–h were prepared by the Erlenmeyer–Plöchl synthesis [14,15] through reaction of hippuric acid 1 with different benzaldehyde derivatives 2a–g or 5-methylfurfural 2h in acetic anhydride in the presence of Hünig’s base [16]. Then, the target diamide-based benzenesulfonamides 5a–h were obtained via reaction of azlactone intermediates 3a–h with *p*-aminobenzenesulfonamide 4 in glacial acetic acid in the presence of sodium acetate to afford the corresponding target benzenesulfonamides 5a–h with 80–93% yield (Scheme 1).

The elemental and spectral data supported the structures of the target benzenesulfonamides 5a–h. IR spectra of 5a–h revealed the presence of bands for (NH_2_) group at 3316–3249 cm^−1^, and bands of (C=O) group around 1629–1636 cm^−1^. Also, their IR spectra displayed two bands of (SO_2_) at (1350–1358) and (1157–1168) cm^−1^. Furthermore, the ^1^H NMR spectra of benzenesulfonamides 5a–h displayed singlet signal of D_2_O exchangeable NH_2_ protons of salfamoyl group at *δ* 7.25–7.49 *ppm*. In addition to presence of olifenic signal at *δ* 7.04–7.32 *ppm*, and two D_2_O exchangeable NH protons at *δ* 9.85–10.16 *ppm* and *δ* 10.23–10.65 *ppm.* On the other hand, compounds 5d–g were confirmed by presence of aliphatic signals of; CH_3_ protons at *δ* 2.28–2.36 *ppm* for 5d, OCH_3_ protons at *δ* 3.74–3.83 *ppm* for 5e–g. Moreover, ^13^C NMR spectra of compounds 5a–h displayed two signals for C=O groups at *δ* 164.52–169.74 *ppm*, also, ^13^C NMR spectra for 5d–g displayed the aliphatic signals of CH_3_ at *δ* 21.38–21.75 *ppm* for 5d, and OCH_3_ at *δ* 55.52–56.11 *ppm* for 5f and 5g.

### 2.2. Biological Evaluation 

#### 2.2.1. Carbonic Anhydrase Inhibition

The CA inhibitory activity of all the newly prepared diamide-based benzenesulfonamides 5a–h was evaluated towards the cytosolic isoforms hCA I and II, as well as towards the transmembrane tumor-associated isoforms hCA IX and XII using an applied photophysics stopped-flow instrument for assaying the CA-catalyzed CO_2_ hydration activity [17]. The inhibitory activities were compared to acetazolamide (AAZ), a clinically used standard CA inhibitor. The following SAR could be derived from the results in Table 1:

(i) The cytosolic isoform hCA I was weekly inhibited by most of the diamide-based benzenesulfonamides 5a–h with inhibition constants (*K*_I_s) in the micromolar range, in detail, between 1.006 and 8.175 μM, except for the *p*-tolyl analogue 5d which arose as a nanomolar hCA I inhibitor with a *K*_I_ equals 796.1 nM, that represents 3-fold decreased activity to the reference drug AAZ (*K*_I_ = 250 nM).

(ii) The physiologically dominant isoform hCA II was moderately inhibited by the target diamides 5a–h with *K*_I_s spanning in the nanomolar range: 68.3–448 nM, Table 1. The SAR outcomes hinted that mono substitution of the benzylidene moiety with 4-Cl or 4-CH_3_ substituents (compounds 5a and 5d; *K*_I_s = 68.3 and 94.4 nM, respectively) is more beneficial for the activity against hCA II than di-substitution with Cl or OCH_3_ substituents (compounds 5b, 5f and 5g; *K*_I_s = 223.9, 294.2 and 448.0 nM, respectively). 

(iii) The target tumor-associated isoform hCA IX was efficiently inhibited by the prepared diamide-based benzenesulfonamides 5a–h (*K*_I_ values ranging between 8.3 and 78 nM, Table 1), apart from the 3,4-(OCH_3_)_2_ benzylidene-bearing sulfonamide 5g, which possessed a slightly reduced inhibitory activity (*K*_I_ = 123.3 nM). Notably, diamides 5a and 5h stood out as a single-digit nanomolar hCA IX inhibitors with *K*_I_ values = 8.8 and 8.3 nM, respectively, which are about three times more potent than the standard drug AAZ (*K*_I_ = 25 nM). Besides, diamide 5b (*K*_I_ = 18.3 nM) was 1.4 times more active than AAZ.

Regarding the impact of substitution of the benzylidene moiety, the hCA IX inhibitory activities were decreased in the following order: 4-Cl > 2,4-(Cl)_2_ > 4-Br > 4-CH_3_ > 2,4-(OCH_3_)_2_ > 4-OCH_3_ > 3,4-(OCH_3_)_2_. This order of activity highlighted that incorporation of the electron-withdrawing halogens (compounds 5a–c; *K*_I_s: 8.8–33.5 nM) is more advantageous for the inhibitory activity toward hCA IX than the electron-donating methyl or methoxy groups (compounds 5d–g; *K*_I_s: 62.1–123.3 nM).

It is noteworthy that bioisosteric replacement of the substituted benzylidene moiety (compounds 5a–g; *K*_I_s: 8.8–123.3 nM) with a hetero 2-furylidene moiety (compound 5h; *K*_I_ = 8.3 nM) resulted in a 1.1-fold to 14.9-fold efficacy enhancement, which could be attributed to an extra hydrogen bonding within the active site through the heterocyclic oxygen atom. 

(iv) The data displayed in Table 1 ascribed to the prepared diamide-based benzenesulfonamides good efficacy in inhibiting the transmembrane tumor-associated isoform hCA XII. The inhibition profiles were found to be rather flat, since the measured *K*_I_s ranged between 9.8 and 60.2 nM, aside from diamide 5f whose efficacy raised to slightly higher concentration (*K*_I_ = 134.5 nM). In particular, diamide 5g was emerged as the only single-digit nanomolar hCA XII inhibitor in this study (*K*_I_ = 9.8 nM).

Concerning the effect of substitution of the benzylidene moiety, the hCA XII inhibitory activities were decreased in the order of: 3,4-(OCH_3_)_2_ > 2,4-(Cl)_2_ > 4-Cl > 4-OCH_3_ > 4-Br > 4-CH_3_ > 2,4-(OCH_3_)_2_. It is worth stressing that, that bioisosteric replacement of the substituted benzylidene moiety with the 2-furylidene moiety elicited a worsening of effectiveness against hCA XII, unlike arose for hCA IX.

(v) Exploration of the the CA inhibitory trends in Table 1 revealed that all the examined diamide-based benzenesulfonamides 5a–h displayed interesting selectivity towards hCA IX over hCA I spanning in the range 12.8–985 (Table 2). Also, the examined diamides possessed good hCA IX/II selectivity indexes spanning in the range 3.6–13.8, apart from diamides 5d and 5e which displayed modest selectivity (SI = 1.4 and 1.5, respectively).

Indeed, it is worth highlighting that bioisosteric replacement of the benzylidene moiety with the hetero 2-furylidene moiety, compound 5h, achieved the best IX/I and IX/II selectivity herein reported with SIs of 985 and 13.8, respectively.

#### 2.2.2. Antitumor Activity 

##### Antitumor Activity towards 60 Cancer Cell Lines (NCI, USA)

First, all diamide-based benzenesulfonamides herein reported 5a–h were screened for their antitumor activity at one dose (10^−5^ M) assay against a panel of sixty cancer cell lines through the National Cancer Institute (NCI) Developmental Therapeutic Program (www.dtp.nci.nih.gov), according to US-NCI protocol. The cell growth and cell viability were evaluated using the sulforhodamine B (SRB) colorimetric assay [18,19,20].

**Figure 2:**
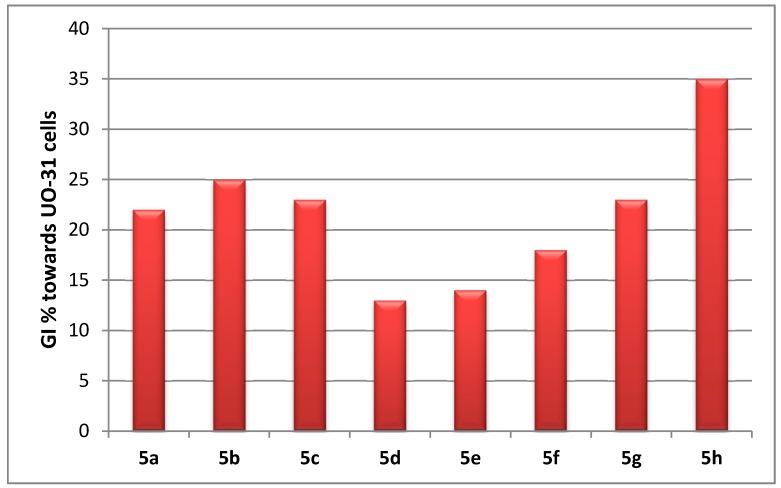
Percentage growth inhibition (GI%) for the target diamide-based benzenesulfonamides **5a**–**h** towards the renal cancer UO-31 cell line.

The obtained data were reported as mean-graph of the percentage growth of the different treated tumour cells (Supplementary Materials). Exploration of results for this assay confirmed that the examined diamide-based benzenesulfonamides 5a–h had non-significant activity towards most NCI cancer cell lines. Of particular interest, all the prepared diamides displayed interesting selectivity towards renal cancer UO-31 cell line with percentage growth inhibition (GI%) range of 13–35% (Figure 2). Compound 5h was found to be the most potent member against UO-31 cells, in this assay, with GI% equals 35. 

##### Anti-Proliferative Activity towards Renal Cancer UO-31 Cell Line

As diamide-based benzenesulfonamides 5a, 5b and 5h emerged as low-nanomolar hCA IX inhibitors (*K*_I_s: of 8.3–18.3 nM) with good hCA IX/I and IX/II selectivity (SI: 326.6–985 and 7.8–13.8, respectively), they were selected to be evaluated for their antitumor activity against renal cancer UO-31 cell line, using the protocol of MTT colorimetric assay as reported by T. Mosmann [21]. Staurosporine was adopted in the experiment as a reference anticancer drug. The results are presented as IC_50_ values and displayed in Table 3.

The results for the MTT assay presented in Table 3 revealed that that the examined diamides possessed good anti-proliferative activity towards UO-31 cell line (IC_50_ range: 4.89 ± 0.22–16.68 ± 0.92 μM). Notably, diamide-based sulfonamide 5h was the most potent derivative with about 1.5-fold more enhanced activity (IC_50_ = 4.89 ± 0.22 μM) than the reference drug Staurosporine (IC_50_ = 7.25 ± 0.43 μM). Also, compound 5a showed comparable activity (IC_50_ = 6.53 ± 0.38 μM) to the reference drug Staurosporine. 

##### Cell Cycle Analysis

**Figure 3:**
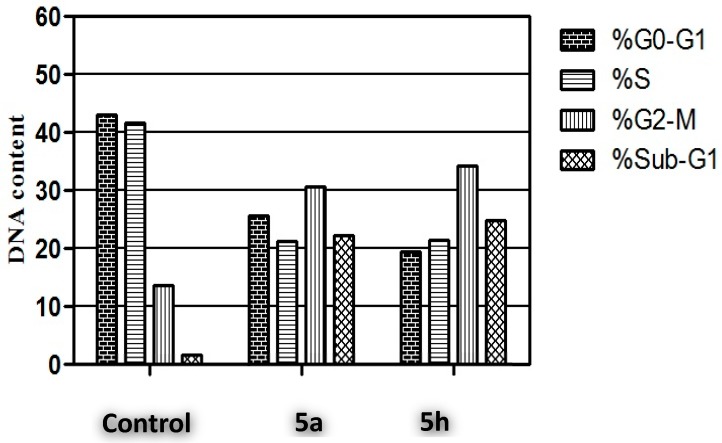
Effect of diamide-based benzenesulfonamides 5a and 5h on the phases of cell cycle of UO-31 cells.

The effect of diamide-based benzenesulfonamides 5a and 5h on cell cycle progression was examined in renal UO-31 cancer cells, through a DNA flow cytometric assay (Figure 3). The UO-31 cells were treated with diamides 5a and 5h at their IC_50_ concentrations (IC_50_ = 6.53 ± 0.38 and 4.89 ± 0.22 µM) for 24 h.

The outcomes of this flow cytometric assay (Figure 3) suggested that exposure of renal UO-31 cells to diamides 5a and 5h led to a significant increase in the percentage of cells at Sub-G_1_ by 14- and 15.5-folds, respectively, with concurrent significant arrest in the G_2_-M phase by 2.2- and 2.5-folds, respectively, compared to control, which could be considered as significant remarks for sulfonamides 5a and 5h to provoke apoptosis in renal UO-31 cells.

##### Annexin V-FITC Apoptosis Assay

**Figure 4:**
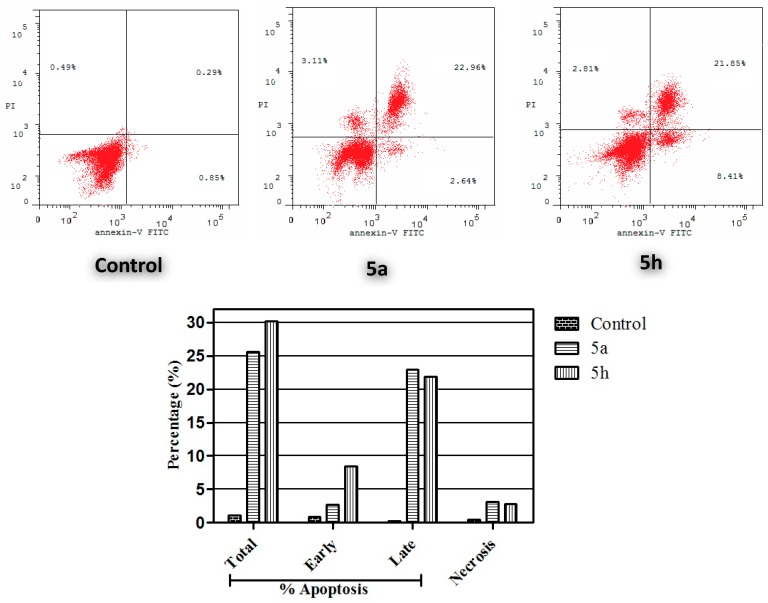
Effect of diamide-based benzenesulfonamides 5a and 5h on the percentage of annexin V-FITC-positive staining in UO-31 cells. The experiments were done in triplicates. The four quadrants identified as: LL, viable; LR, early apoptotic; UR, late apoptotic; UL, necrotic.

To explore whether the anti-proliferative activity of diamide-based sulfonamides 5a and 5h is consistent with the induction of apoptosis within the UO-31 cancer cells, Annexin V-FITC/propidium iodide (AV/PI) dual staining assay was performed by flow cytometry (Figure 4). 

The results suggested that diamides 5a and 5h could induce the apoptosis of UO-31 cells as evidenced by the significant increase in the percent of annexin V-FITC-positive apoptotic cells, including both the early (from 0.85% to 2.64% for 5a, and from 0.95% to 8.41% for 5h) and late apoptotic (from 0.29% to 22.96% for 5a, and from 0.29% to 21.85% for 5h) phases (UR + LR), which represents about 22.5- and 26.5-folds total increase as compared with the control. 

### 2.3. Molecular Modelling Study 

To provide insights into the binding interactions of the reported diamide-based benzenesulfonamides within hCA isozymes II and IX (PDB 5LJT [22] and 5FL4 [23]), docking and MM-GBSA-based refinements were performed. As expected, in all docking solutions found for compounds 5a–h, the benzenesulfonamide is placed deeply into the active site region of both isozymes, with the zinc-binding group coordinating to the metal ion through its negatively charged nitrogen. Moreover, the sulfonamide moiety engages two H-bonds: the NH^–^ group acts as donor, while the S=O as acceptor with T199 OG1 atom and backbone NH respectively. The phenyl ring accommodates in the hydrophobic region lined by V121, V143 and L198.

**Figure 5:**
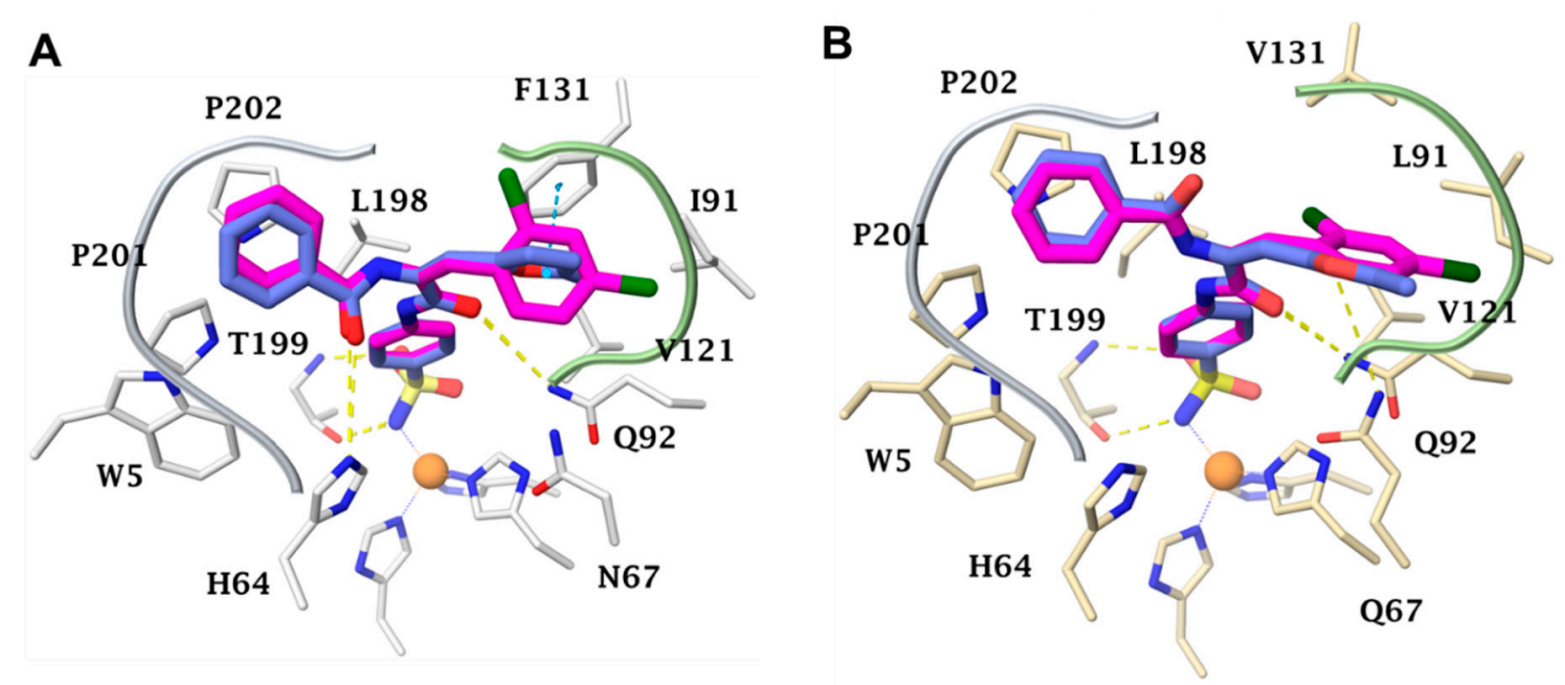
Docking of 5b (purple) and 5h (violet) in hCA II (**A**) and in hCA IX (**B**) active sites.

In hCA II, the carbonyl group of the amidic linker of derivatives 5a–h accepts a H-bond by the HE-Q92, orienting the arylidene moiety in a cleft defined by N67, Q92, I91 and F131 residues, where compounds 5a–g can engage π-π interactions. The benzoyl moiety accessed in a lipophilic pocket situated just above the H64, whose imidazole NH acts as H-bond donor towards the oxygen atom of the carbonyl group (Figure 5A).

As for hCA II, a similar set of interactions is also found for the hCA IX isoform. However, because of the substitution V131/F131 (hCA XI/II), the arylidene moiety accommodates in a wider cavity, more suited to the size of the group. As a result, the latter makes more effective interactions with the receptor counterpart, thus probably contributing to the 1.5 to 14-fold hCA IX/II selectivity of compounds 5a–h. The methylfuryl derivative 5h further shows an additional H-bond between the heterocyclic oxygen and Q67 HE atom (Figure 5B).

## 3. Materials and Methods

### 3.1. Chemistry

#### 3.1.1. General

Melting points were measured with a Stuart melting point apparatus and were uncorrected. The NMR spectra were recorded by Varian Gemini-400BB 400 MHz FT-NMR spectrometers (Varian Inc., Palo Alto, CA). ^1^H and ^13^C spectra were run at 400 and 100 MHz, respectively, in deuterated dimethylsulphoxide (DMSO-*d_6_*). All coupling constant (*J*) values are given in hertz. Chemical shifts (*δ*_C_) are reported relative to DMSO-*d_6_* as internal standards. The abbreviations used are as follows: s, singlet; d, doublet; m, multiplet. IR spectra were recorded with a Bruker FT-IR spectrophotometer. Reaction courses and product mixtures were routinely monitored by thin layer chromatography (TLC) on silica gel precoated F_254_ Merck plates. Unless otherwise noted, all solvents and reagents were commercially available and were used without further purification. Azlactones 3a–g were previously reported [24,25].

#### 3.1.2. General Procedure for Preparation of Target Diamide-Based Benzenesulfonamides 5a–h

A mixture of *p*-aminobenzenesulfonamide 4 (0.17 g, 1 mmol), sodium acetate (0.08 g, 1 mmol) and the appropriate azlactone derivative 3a–h (1 mmol) in glacial acetic acid was heated under reflux for 4 h. The solid product obtained upon cooling was filtered off and recrystallized from acetone to produce the corresponding benzenesulfonamides 5a–h, with 80–93% yield.

##### N-(1-(4-Chlorophenyl)-3-oxo-3-((4-sulfamoylphenyl)amino)prop-1-en-2-yl)benzamide (5a)

White crystal (yield 93%), m.p. 255–258 °C; IR (KBr, ν cm^−1^): 3311, 3255 (NH, NH_2_), 1704, 1639 (2C=O) and 1316, 1156 (SO_2_); ^1^H NMR (DMSO-*d6*) *δ ppm*: 7.10 (s, 1H, olefinic), 7.24 (s, 2H, NH_2_ D_2_O exchangeable), 7.44 (d, 2H, *J* = 8.4 Hz, H-3, H-5 of C_6_H_5_), 7.49 (t, 1H, *J* = 8.0 Hz, H-4 of C_6_H_5_), 7.51 (d, 2H, *J* = 8.0 Hz, H-3, H-5 of 4-Cl-C_6_H_4_), 7.62 (d, 2H, *J* = 8.4 Hz, H-2, H-6 of C_6_H_5_), 7.74 (d, 2H, *J* = 8.8 Hz, H-2, H-6 of sulfonamide), 7.85 (d, 2H, *J* = 8.8 Hz, H-3, H-5 of sulfonamide), 7.98 (d, 2H, *J* = 8.0 Hz, H-2, H-6 of 4-Cl-C_6_H_4_), 10.15 (s, 1H, NH D_2_O exchangeable), 10.53 (s, 1H, NH D_2_O exchangeable); ^13^C NMR (DMSO-*d*_6_) *δ ppm*: 119.93, 126.92, 127.37, 128.39, 128.88, 129.08, 131.60, 131.93, 132.39, 133.57, 133.60, 133.66, 138.97, 142.70, 165.13, 166.48; HRMS (ESI) for C_22_H_19_ClN_3_O_4_S, calcd 456.07793, found 456.07800 [M+H]^+^; Anal. calcd. for C_22_H_18_ClN_3_O_4_S (455.91): C, 57.96; H, 3.98; N, 9.22. Found C, 58.17; H, 3.89; N, 9.11.

##### N-(1-(2,4-Dichlorophenyl)-3-oxo-3-((4-sulfamoylphenyl)amino)prop-1-en-2-yl)benzamide (5b)

White crystal (yield 91%), m.p. 265–267 °C; IR (KBr, ν cm^−1^): 3303, 3249 (NH, NH_2_), 1711, 1629 (2C=O) and 1310, 1153 (SO_2_); ^1^H NMR (DMSO-*d6*) *δ ppm*: 7.04 (s, 1H, olefinic), 7.25 (s, 2H, NH_2_ D_2_O exchangeable), 7.40 (dd, 1H, *J* = 2.0 Hz, *J* = 8.4 Hz, H-5 of 2,4(Cl)_2_-C_6_H_3_), 7.46 (t, 1H, *J* = 8.0 Hz, H-4 of C_6_H_5_), 7.48 (d, 1H, *J* = 8.0 Hz, H-6 of 2,4(Cl)_2_-C_6_H_3_), 7.55 (d, 2H, *J* = 8.0 Hz, H-3, H-5 of C_6_H_5_), 7.71 (s, 1H, H-3 of 2,4(Cl)_2_-C_6_H_3_), 7.75 (d, 2H, *J* = 8.8 Hz, H-2, H-6 of sulfonamide), 7.86 (d, 2H, *J* = 8.8 Hz, H-3, H-5 of sulfonamide), 7.91 (d, 2H, *J* = 7.6 Hz, H-2, H-6 of C_6_H_5_), 10.14 (s, 1H, NH D_2_O exchangeable), 10.57 (s, 1H, NH D_2_O exchangeable); ^13^C NMR (DMSO-*d*_6_) *δ ppm*: 120.08, 122.58, 126.95, 127.98, 128.41, 128.83, 129.50, 131.53, 132.19, 132.45, 133.54, 133.95, 134.06, 134.49, 139.18, 142.44, 164.52, 166.46; Anal. calcd. for C_22_H_17_Cl_2_N_3_O_4_S (490.36): C, 53.89; H, 3.49; N, 8.57. Found C, 53.65; H, 3.48; N, 8.68.

##### N-(1-(4-Bromophenyl)-3-oxo-3-((4-sulfamoylphenyl)amino)prop-1-en-2-yl)benzamide (5c)

Yellow powder (yield 80%), m.p. 210–212 °C; IR (KBr, ν cm^−1^): 3312, 3250 (NH, NH_2_), 1704, 1636 (2C=O) and 1316, 1156 (SO_2_); ^1^H NMR (DMSO-*d6*) *δ ppm*: 7.07 (s, 1H, olefinic), 7.29 (s, 2H, NH_2_ D_2_O exchangeable), 7.42 (t, 1H, *J* = 8.0 Hz, H-4 of C_6_H_5_), 7.45- 7.52 (m, 2H, H-3, H-5 of C_6_H_5_), 7.52–7.58 (m, 2H, H-3, H-5 of 4-Br-C_6_H_4_), 7.70, 8.28 (d, 2H, H-2, H-6 of C_6_H_5_), 7.74 (d, 2H, H-2, H-6 of sulfonamide), 7.85 (d, 2H, H-3, H-5 of sulfonamide), 7.98 (d, 2H, *J* = 8.0 Hz, H-2, H-6 of 4-Br-C_6_H_4_), 10.61 (s, 2H, NH D_2_O exchangeable); ^13^C NMR (DMSO-*d*_6_) *δ ppm*: 119.89, 122.28, 124.82, 126.91, 126.99, 127.07, 127.14, 127.51, 128.23, 128.40, 128.65, 128.79, 128.84, 128.93, 129.02, 129.46, 130.49, 131.65, 131.83, 131.98, 132.20, 132.22, 132.32, 132.43, 133.69, 133.77, 133.98, 134.52, 137.59, 138.90, 139.18, 142.76, 144.17, 161.30, 165.24, 166.52, 169.69; Anal. calcd. for C_22_H_18_BrN_3_O_4_S (500.37): C, 52.81; H, 3.63; N, 8.40. Found C, 60.02; H, 3.60; N, 8.31.

##### N-(3-Oxo-3-((4-sulfamoylphenyl)amino)-1-(p-tolyl)prop-1-en-2-yl)benzamide (5d)

Yellow powder (yield 87%), m.p. 255–258 °C; IR (KBr, ν cm^−1^): 3313, 3251 (NH, NH_2_), 1701, 1639 (2C=O) and 1319, 1157 (SO_2_); ^1^H NMR (DMSO-*d6*) *δ ppm*: 2.28, 2.36 (2s, H, CH_3_), 7.13, 7.24 (2s, 1H, olefinic), 7.27 (s, 2H, NH_2_ D_2_O exchangeable), 7.18, 8.00 (2d, 2H, *J* = 7.6, H-3, H-5 of C_6_H_5_), 7.31, 8.23 (2d, 2H, *J* = 8.4 Hz, H-2, H-6 of C_6_H_5_), 7.39–7.51 (m, 4H, Ar-H of 4-CH_3_-C_6_H_4_), 7.49, 7.53 (2t, 1H, *J* = 8.0 Hz, H-4 of C_6_H_5_), 7.74 (d, 2H, H-2, H-6 of sulfonamide), 7.85 (d, 2H, H-3, H-5 of sulfonamide), 10.09 (s, 1H, NH D_2_O exchangeable), 10.45 (s, 1H, NH D_2_O exchangeable); ^13^C NMR (DMSO-*d*_6_) *δ ppm*: 21.38, 21.75 (CH_3_), 119.95, 126.88, 127.10, 128.34, 128.60, 128.85, 128.96, 129.01, 129.38, 129.63, 130.01, 130.07, 130.36, 131.72, 131.77, 131.97, 132.28, 132.92, 133.85, 137.74, 137.93, 138.86, 139.02, 141.51, 142.77, 144.04, 160.23, 165.31, 166.44, 169.74; HRMS (ESI) for C_23_H_22_N_3_O_4_S, calcd 436.13255, found 436.13251 [M+H]^+^; Anal. calcd. for C_23_H_21_N_3_O_4_S (435.50): C, 63.43; H, 4.86; N, 9.65. Found C, 63.57; H, 4.83; N, 9.58.

##### N-(1-(4-Methoxyphenyl)-3-oxo-3-((4-sulfamoylphenyl)amino)prop-1-en-2-yl)benzamide (5e)

Yellow powder (yield 88%), m.p. 265–268 °C; IR (KBr, ν cm^−1^): 3309, 3247 (NH, NH_2_), 1703, 1634 (2C=O) and 1314, 1154 (SO_2_); ^1^H NMR (DMSO-*d6*) *δ ppm*: 3.74, 3.83 (2s, 3H, OCH_3_), 6.93, 8.33 (2d, 2H, *J* = 8.8 Hz, H-3, H-5 of C_6_H_5_), 7.07, 8.04 (2d, 2H, *J* = 8.4 Hz, H-2, H-6 of C_6_H_5_), 7.18, 7.32 (2s, 1H, olefinic), 7.39–7.45 (m, 4H, H-3, H-5 and H-2, H-6 of 4-OCH_3_-C_6_H_4_), 7.47 (t, 1H, *J* = 8.0 Hz, H-4 of C_6_H_5_), 7.49 (s, 2H, NH_2_ D_2_O exchangeable), 7.54, 7.72 (2d, 2H, *J* = 8.8 Hz, H-2, H-6 of sulfonamide), 7.58, 7.85 (2d, 2H, *J* = 8.8 Hz, H-3, H-5 of sulfonamide), 10.95 (s, 2H, NH D_2_O exchangeable); Anal. calcd. for C_23_H_21_N_3_O_5_S (451.50): C, 61.19; H, 4.69; N, 9.31. Found C, 60.88; H, 4.65; N, 9.30.

##### N-(1-(2,4-Dimethoxyphenyl)-3-oxo-3-((4-sulfamoylphenyl)amino)prop-1-en-2-yl)benzamide (5f)

Yellow powder (yield 85%), m.p. 245–250 °C; IR (KBr, ν cm^−1^): 3410, 3294 (NH, NH_2_), 1701, 1639 (2C=O) and 1369, 1161 (SO_2_); ^1^H NMR (DMSO-*d6*) *δ ppm*: 3.75 (s, 3H, OCH_3_), 3.81 (s, 3H, OCH_3_), 6.65 (s, 1H, H-3 of (OCH_3_)_2_-C_6_H_3_), 7.42 (dd, 2H, *J* = 2.4 Hz, *J* = 9.2 Hz, H-5, H-6 of (OCH_3_)_2_-C_6_H_3_), 7.41–7.47 (m, 4H, H-3, H-5 of C_6_H_4_ and NH_2_ D_2_O exchangeable), 7.49 (t, 1H, *J* = 8.0 Hz, H-4 of C_6_H_5_), 7.55 (s, 1H, olefinic), 7.63 (d, 2H, H-2, H-6 of C_6_H_5_), 7.72 (d, 2H, *J* = 8.8 Hz, H-2, H-6 of sulfonamide), 7.85 (d, 2H, *J* = 8.8 Hz, H-3, H-5 of sulfonamide), 10.16 (s, 1H, NH D_2_O exchangeable), 10.65 (s, 1H, NH D_2_O exchangeable); Anal. calcd. for C_24_H_23_N_3_O_6_S (481.52): C, 59.87; H, 4.81; N, 8.73. Found C, 60.09; H, 4.83; N, 8.67.

##### N-(1-(3,4-Dimethoxyphenyl)-3-oxo-3-((4-sulfamoylphenyl)amino)prop-1-en-2-yl)benzamide (5g)

Yellow powder (yield 90%), m.p. 250–253 °C; IR (KBr, ν cm^−1^): 3413, 3292 (NH, NH_2_), 1701, 1639 (2C=O) and 1369, 1161 (SO_2_); ^1^H NMR (DMSO-*d6*) *δ ppm*: 3.75 (s, 3H, OCH_3_), 3.78 (s, 3H, OCH_3_), 6.97, 8.20 (d, 2H, H-3, H-5 of C_6_H_5_), 7.21–7.29 (m, 4H, H-5, H-6 of (OCH_3_)_2_-C_6_H_3_ and NH2 D_2_O exchangeable), 7.43–7.46 (m, 2H, H-3 of (OCH_3_)_2_-C_6_H_3_ and olefinic), 7.48, 7.51 (t, 1H, *J* = 8.0 Hz, H-4 of C_6_H_5_), 7.57, 8.04 (d, 2H, *J* = 8.4 Hz, H-2, H-6 of C_6_H_5_), 7.74 (d, 2H, *J* = 8.8 Hz, H-2, H-6 of sulfonamide), 7.86 (d, 2H, *J* = 8.8 Hz, H-3, H-5 of sulfonamide), 10.07 (s, 1H, NH D_2_O exchangeable), 10.36 (s, 1H, NH D_2_O exchangeable); ^13^C NMR (DMSO-*d*_6_) *δ ppm*: 55.52 (OCH_3_), 55.95 (OCH_3_), 112.00, 112.95, 120.07, 124.11, 126.86, 127.02, 127.11, 128.31, 128.57, 128.75, 128.82, 129.04, 129.22, 130.30, 132.31, 133.83, 138.84, 142.77, 148.73, 150.03, 165.28, 166.37; Anal. calcd. for C_24_H_23_N_3_O_6_S (481.52): C, 59.87; H, 4.81; N, 8.73. Found C, 60.13; H, 4.77; N, 8.64.

##### N-(1-(5-Methylfuran-2-yl)-3-oxo-3-((4-sulfamoylphenyl)amino)prop-1-en-2-yl)benzamide (5h)

Orange powder (yield 87%), m.p. 240–243 °C; IR (KBr, ν cm^−1^): 3413, 3295 (NH, NH_2_), 1706, 1636 (2C=O) and 1366, 1160 (SO_2_); ^1^H NMR (DMSO-*d6*) *δ ppm*: 2.20, 2.40 (2s, 1H, CH_3_), 6.23 (d, 1H, H-4 furan), 6.46, 8.13 (2d, 2H, H-3, H-5 of C_6_H_5_), 6.69 (d, 1H, H-3 furan), 7.05, 7.12 (2s, 1H, olefinic), 7.23 (s, 2H, NH_2_ D_2_O exchangeable), 7.33–7.47 (m, 2H, H-2, H-6 of C_6_H_5_), 7.48 (t, 1H, *J* = 8.0 Hz, H-4 of C_6_H_5_), 7.74 (d, 2H, H-2, H-6 of sulfonamide), 7.83–7.86 (m, 2H, H-3, H-5 of sulfonamide), 9.85 (s, 1H, NH D_2_O exchangeable), 10.23 (s, 1H, NH D_2_O exchangeable); ^13^C NMR (DMSO-*d*_6_) *δ ppm*: 13.89 (CH_3_), 14.30 (CH_3_), 109.31, 111.59, 115.59, 116.81, 118.58, 120.17, 121.62, 122.08, 122.46, 126.32, 126.83, 127.06, 127.10, 128.34, 128.71, 128.93, 128.99, 129.02, 129.31, 131.42, 131.83, 132.13, 134.60, 135.05, 137.78, 138.91, 142.61, 144.01, 148.66, 149.53, 149.74, 154.60, 156.33, 157.81, 158.18, 158.88, 164.41, 166.35, 166.43, 169.07; Anal. calcd. for C_21_H_19_N_3_O_5_S (425.46): C, 59.28; H, 4.50; N, 9.88. Found C, 59.15; H, 4.46; N, 9.97.

### 3.2. Biological Evaluation 

#### 3.2.1. CA Inhibitory Assay

An Applied Photophysics stopped-flow instrument was used for assaying the CA catalysed CO_2_ hydration activity, as reported earlier [17]. The inhibition constants were obtained by non-linear least-squares methods using PRISM 3 and the Cheng-Prusoff equation as reported earlier, and represent the mean from at least three different determinations. The four tested CA isofoms were recombinant ones obtained in-house as reported earlier [26,27,28,29].

#### 3.2.2. Anticancer Activity towards 60 Cancer Cell Lines (NCI, Bethesda, MD, USA)

This assay was carried out according to the protocol of the Drug Evaluation Branch, NCI, Bethesda [30,31,32]. The cell growth and cell viability were evaluated using the sulforhodamine B (SRB) [26] colorimetric assay, as reported earlier [33,34].

#### 3.2.3. Antiproliferative Activity towards Renal Cancer UO-31 Cell Line 

Renal cancer UO-31 cell line was obtained from American Type Culture Collection (ATCC). UO-31 cells were seeded into 96-well plates at 1.8 x 10^4^/well. Cells were grown in DMEM, and supplemented with 10% heat-inactivated fetal bovine serum, 1% L-glutamine (2.5 mM), HEPES buffer (10 mM), 50 μg/mL gentamycin. All cells were maintained at 37 °C in a humidified atmosphere with 5% CO_2_. Cytotoxic activity of diamide-based benzenesulfonamides 5a, 5b and 5h was evaluated following the the protocol of MTT colorimetric assay, as reported previously [35,36].

#### 3.2.4. Cell Cycle Analysis

Renal cancer UO-31 cells were treated with diamide-based benzenesulfonamide 5h at its IC_50_ concentration (IC_50_ = 4.89 ± 0.22 µM) for 24 h, and then cells were washed with ice-cold phosphate buffered saline (PBS). Then, the treated cells were collected by centrifugation, fixed in ice-cold 70% (*v*/*v*) ethanol, washed with PBS, re-suspended with RNase (100 μg/mL), stained with PI (40 μg/mL), and analyzed by flow cytometry using FACS Calibur (Becton Dickinson, BD, USA). The cell cycle distributions were calculated utilizing CellQuest software 5.1 (Becton Dickinson) [37,38].

#### 3.2.5. Annexin V-FITC Apoptosis Assay

Assay of the phosphatidylserine externalization was carried out using Annexin V-FITC/PI apoptosis detection kit (BD Biosciences, San Jose, CA, USA) following the manufacturer’s instructions, as reported previously [39,40]. 

#### 3.2.6. Molecular Docking Simulations

Preparation of the crystal structure of both hCAII (PDB 5LJT [32]) and hCAIX (PDB 5FL4 [33]) was performed using the Protein Preparation Wizard tool implemented in Maestro-Schrödinger suite, assigning bond orders, adding hydrogens, deleting water molecules, and optimizing H-bonding networks [41]. Energy minimization protocol with a root mean square deviation (RMSD) value of 0.30 was applied using an Optimized Potentials for Liquid Simulation (OPLS3) force field. For the simulations with sulfonate derivatives, 5JLT and 5FL4 were prepared adding the zinc-bound water molecule as fourth ligand of the metal tetrahedral coordination sphere. 3D ligand structures were prepared by Maestro [43a] and evaluated for their ionization states at pH 7.4 ± 0.5 with Epik [41b]. OPLS3 force field in Macromodel [41e] was used for energy minimization for a maximum number of 2500 conjugate gradient iteration and setting a convergence criterion of 0.05 kcal mol-1Å-1. The docking grid was centered on the center of mass of the co-crystallized ligands, and was Glide used with default settings. Ligands were docked with the standard precision mode (SP) of Glide [41f] and the best 5 poses of each molecule retained as output. The best pose for each compound, evaluated in terms of coordination, hydrogen bond interactions and hydrophobic contacts, was refined by Prime [41d] with a VSGB solvatation model considering the target flexible within 3Å around the ligand [42,43,44].

## 4. Conclusions

In summary, this study reports the synthesis of novel series of diamide-based benzenesulfonamides 5a–h. The prepared diamides were examined as inhibitors of CA isoforms hCA I, II, IX and XII, using a stopped-flow CO_2_ hydrase assay. All the tested isoforms were inhibited by the diamide-based benzenesulfonamides 5a–h herein reported in variable degrees; hCA I was inhibited with *K*_I_s in the range of 796.1–8175nM, hCA II in the range of 68.3–448 nM; hCA IX in the range of 8.3–123.3 nM, whereas hCA XII in the range of 9.8–134.5 nM. Notably, diamides 5a and 5h stood out as a single-digit nanomolar hCA IX inhibitors with *K*_I_ values = 8.8 and 8.3 nM, respectively, which are about three times more potent than the standard drug AAZ (*K*_I_ = 25 nM). All the examined diamides 5a–h displayed interesting selectivity towards hCA IX over hCA I with SIs spanning in the range 12.8–985. Also, they possessed good hCA IX/II SIs in the range 3.6–13.8, apart from diamides 5d and 5e which exhibited modest selectivity (SI = 1.4 and 1.5, respectively). Interestingly, bioisosteric replacement of the benzylidene moiety with the hetero 2-furylidene moiety, compound 5h, achieved the best IX/I and IX/II selectivity herein reported with SIs of 985 and 13.8, respectively. Molecular docking simulations of the prepared diamides in CA IX active site unveiled the ability of 5h to establish an additional H-bond between the heterocyclic oxygen and HE/Gln67. Moreover, benzenesulfonamides 5a, 5b and 5h were selected to be in vitro evaluated for their antitumor activity against renal cancer UO-31 cell line. Notably, 5h was the most potent derivative with about 1.5-fold more enhanced activity (IC_50_ = 4.89 ± 0.22 μM) than the reference drug Staurosporine (IC_50_ = 7.25 ± 0.43 μM). Furthermore, 5a and 5h were able to induce apoptosis in UO-31 cells as evidenced by the significant increase in the percent of annexinV-FITC positive apoptotic cells by 22.5- and 26.5-folds, respectively, as compared with the control. 

## Figures and Tables

**Table 1:** Inhibition data of human CA isoforms hCA I, II, IX and XII for diamide-based benzenesulfonamides 5a–h, determined by stopped-flow CO_2_ hydrase assay, using acetazolamide (AAZ) as a standard drug.

Comp.	R	R_1_	R_2_	*K*_I_ (nM)*			
				hCA I	hCA II	hCA IX	hCA XII
**5a**	H	H	Cl	3955.7	68.3	8.8	16.1
**5b**	Cl	H	Cl	5977.6	223.9	18.3	10.5
**5c**	H	H	Br	2397.8	251.9	33.5	55.4
**5d**	H	H	CH_3_	796.1	94.4	62.1	60.2
**5e**	H	H	OCH_3_	1006.4	127.7	78.0	42.8
**5f**	OCH_3_	H	OCH_3_	5132.7	294.2	73.7	134.5
**5g**	H	OCH_3_	OCH_3_	2207.7	448.0	123.3	9.8
**5h**	-	-	-	8175.4	114.8	8.3	57.9
**AAZ**	-	-	-	250.0	12.0	25.0	5.7
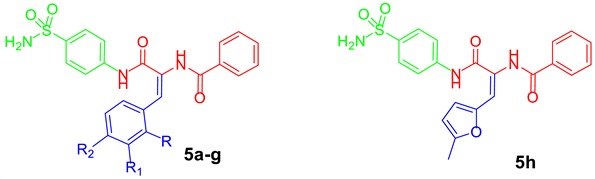							

* Mean from 3 different assays, by a stopped flow technique (errors were in the range of ± 5–10 % of the reported values).

**Table 2:** Selectivity ratios for the inhibition of hCA IX and XII over hCA I and II for diamide-based benzenesulfonamides 5a–h and acetazolamide.

Cmpd	I/IX	II/IX	I/XII	II/XII
5a	449.5	7.8	245.7	4.2
5b	326.6	12.2	569.2	21.3
5c	71.6	7.5	43.3	4.5
5d	12.8	1.5	13.2	1.7
5e	12.9	1.4	23.5	3
5f	69.9	4	38.2	2.2
5g	17.9	3.6	225.3	45.7
5h	985	13.8	141.2	2
AAZ	10.0	0.5	43.9	2.2

**Table 3:** In vitro anti-proliferative activity of diamide-based benzenesulfonamides 5a, 5b and 5h against renal UO-31 cancer cell line.

Compound	IC_50_ (μM) ^a^
	UO-31
5a	6.53 ± 0.38
5b	16.68 ± 0.92
5h	4.89 ± 0.22
Staurosporine	7.25 ± 0.43

**^a^** IC_50_ values are the mean ± S.D. of three separate experiments.

## Supplementary Materials

Supplementary materials can be found at https://www.mdpi.com/1422-0067/20/10/2484/s1.
