# Supplementary material for: Novel Diamide-Based Benzenesulfonamides as Selective Carbonic Anhydrase IX Inhibitors Endowed with Antitumor Activity: Synthesis, Biological Evaluation and In Silico Insights
Source: Int J Mol Sci. 2019 May 20;20(10):2484. doi: 10.3390/ijms20102484 (PMC6566410; doi:10.3390/ijms20102484)

Dr_WagdyMohamed-C-H

Sample Name	Dr_WagdyMohamed-C-H	Pulse sequence	PROTON	Temperature	25	Study owner	vnmr1
Date collected	2016-11-17	Solvent	dmso	Spectrometer	nmr400-mercury400	Operator	vnmr1

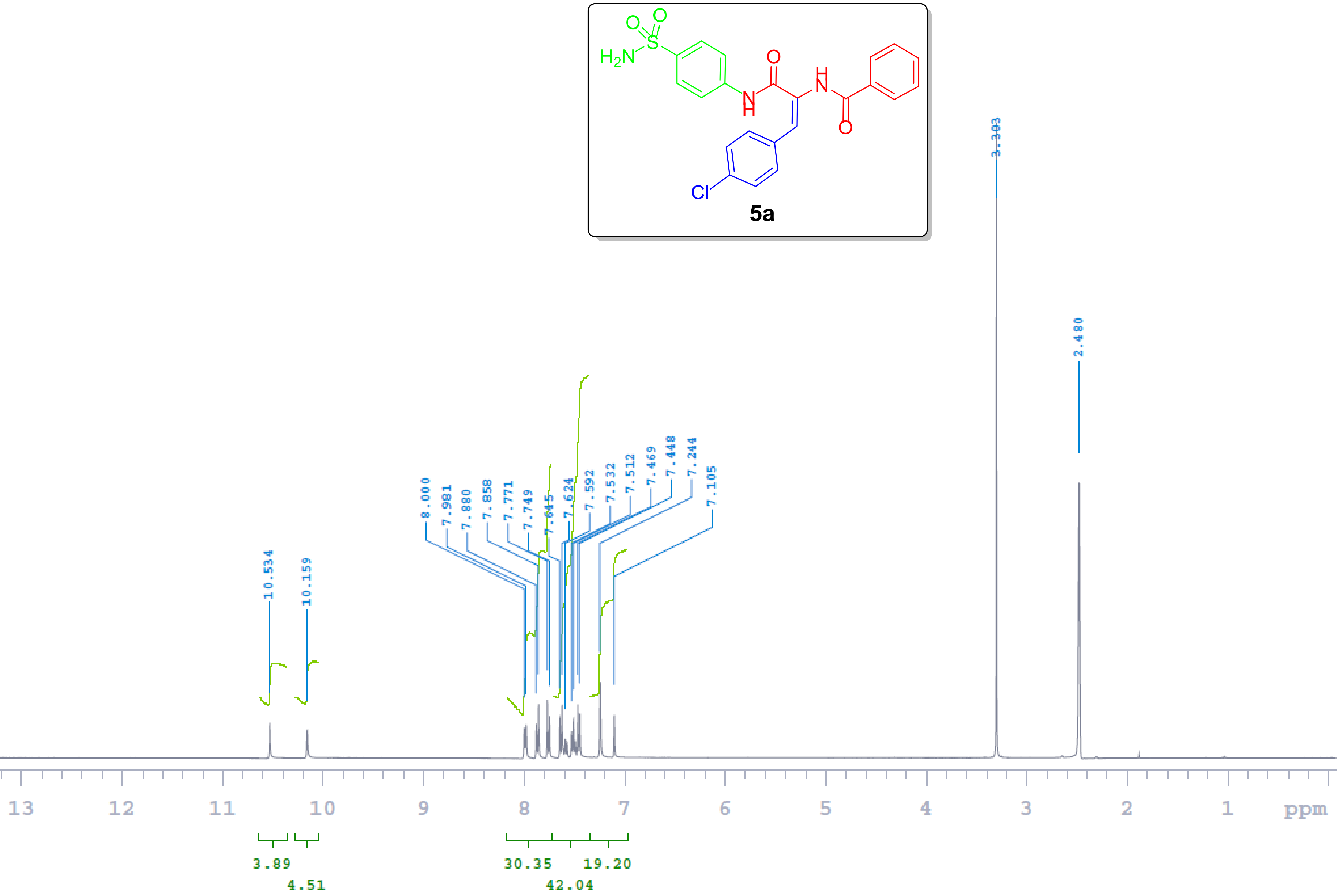

Dr_WagdyMohamed-C-H-D2O

Sample Name	Dr_WagdyMohamed-C-H-D2O	Pulse sequence	PROTON	Temperature	25	Study owner	vnmr1
Date collected	2016-11-20	Solvent	dmso	Spectrometer	nmr400-mercury400	Operator	vnmr1

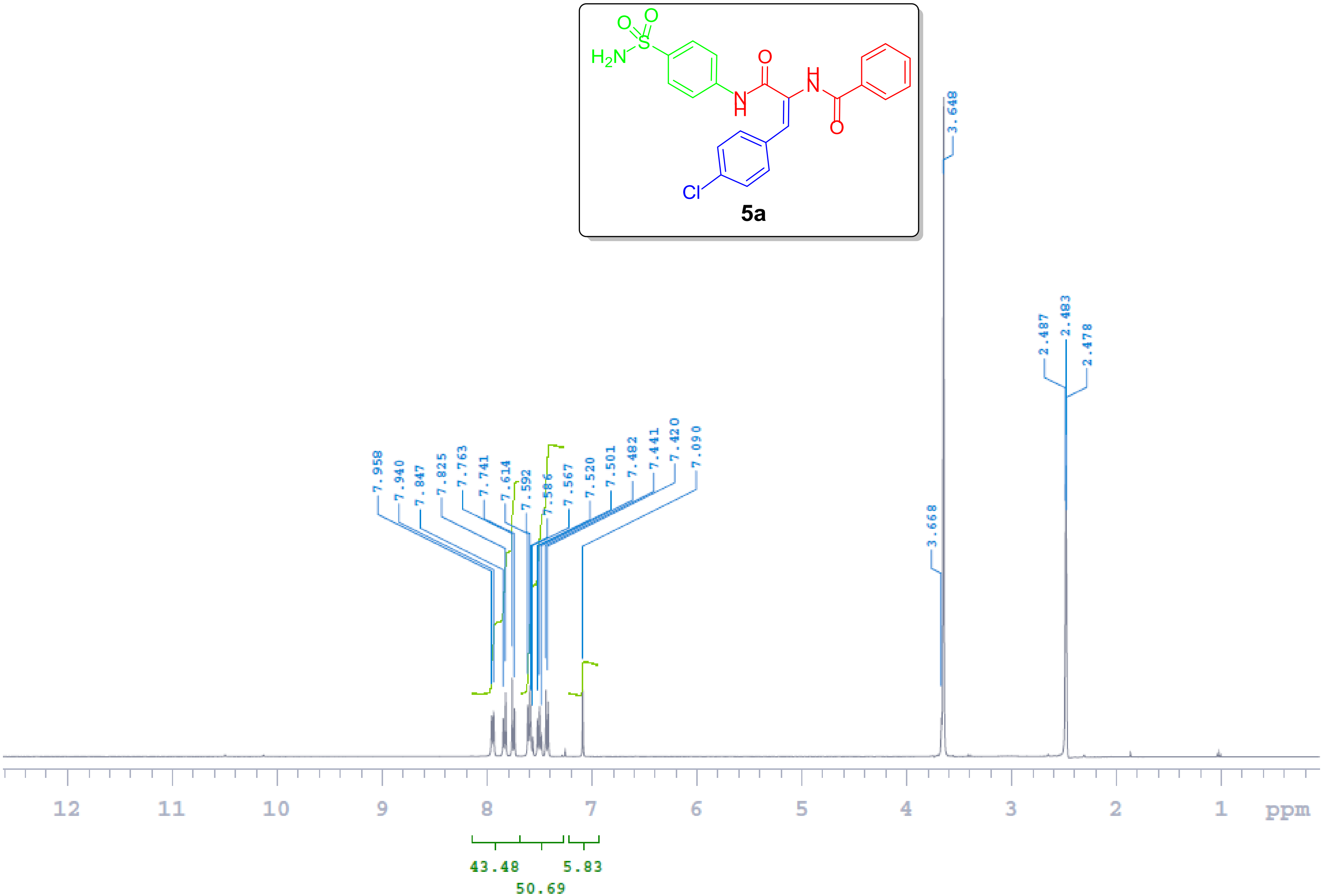

Dr_WagdyMohamed-d_H

Sample Name Dr_WagdyMohamed-d_H | Pulse sequence CARBON | Temperature 25 | Study owner vnmr1
Date collected 2017-01-22 | Solvent dmso | Spectrometer nmr400-mercury400 | Operator vnmr1

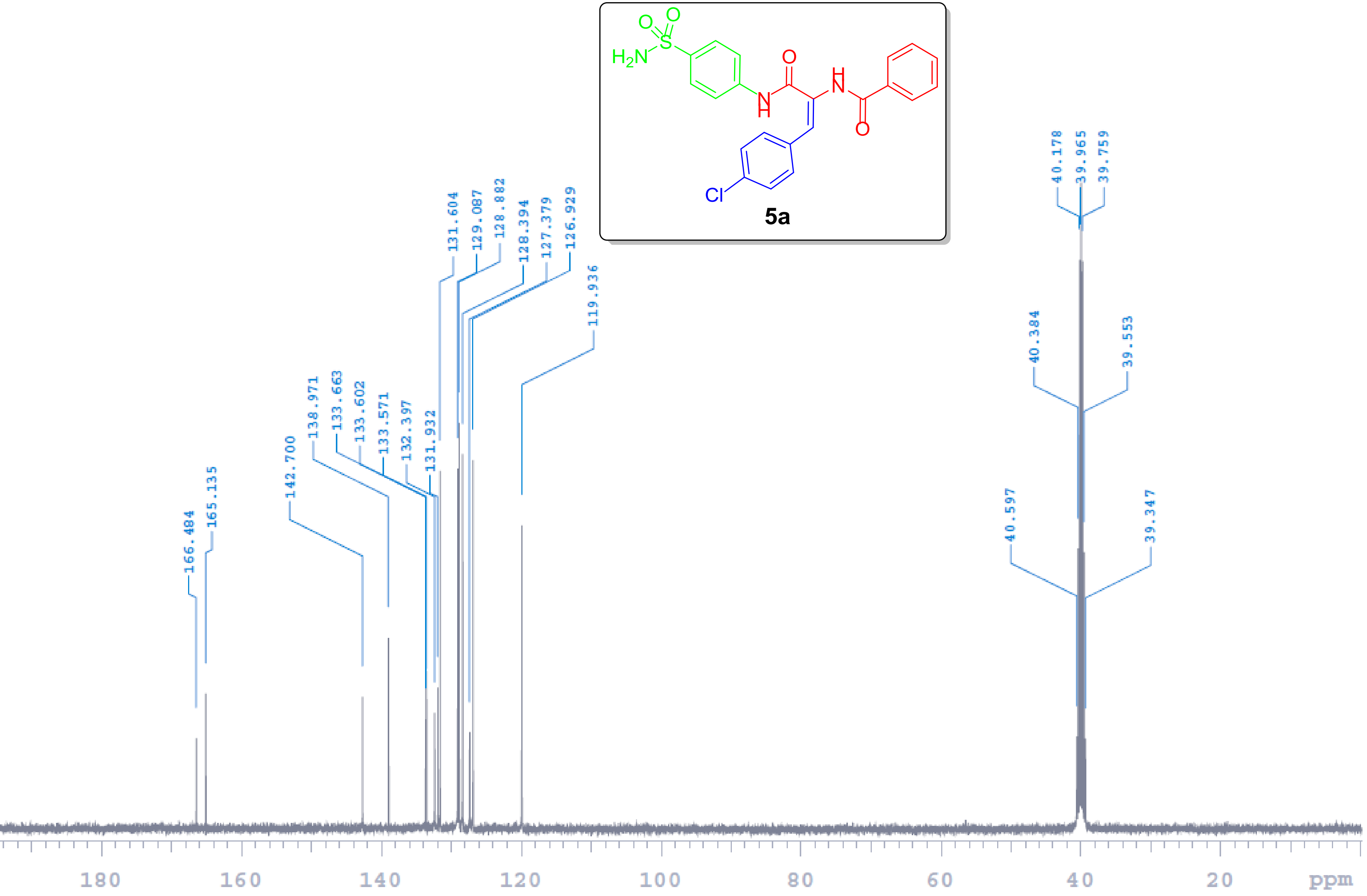

X:\data\Mar 19\ESI75854.raw 27/03/2019 5:05 pm

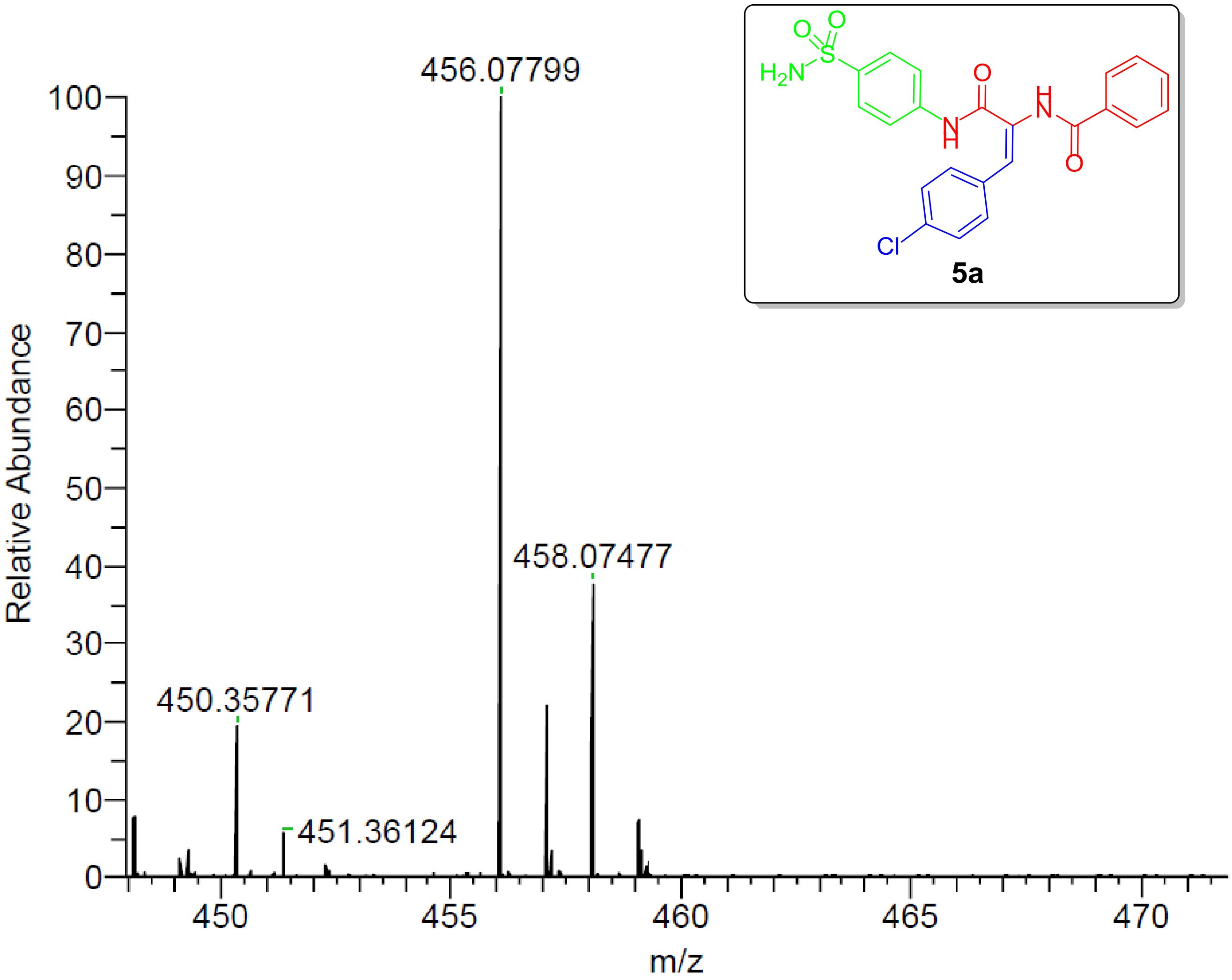

NL: 6.03E5
ESI75854 #13-27 RT: 0.15-0.31 AV: 8 NL: 5.30E+006
T: FTMS {1,1} + p ESI Full lock ms [80.00-1600.00]

Measured Spectrum

Dr_WagdyMohamed-d-H

Sample Name	Dr_WagdyMohamed-d-H	Pulse sequence	PROTON	Temperature	25	Study owner	vnmr1
Date collected	2016-11-17	Solvent	dmso	Spectrometer	nmr400-mercury400	Operator	vnmr1

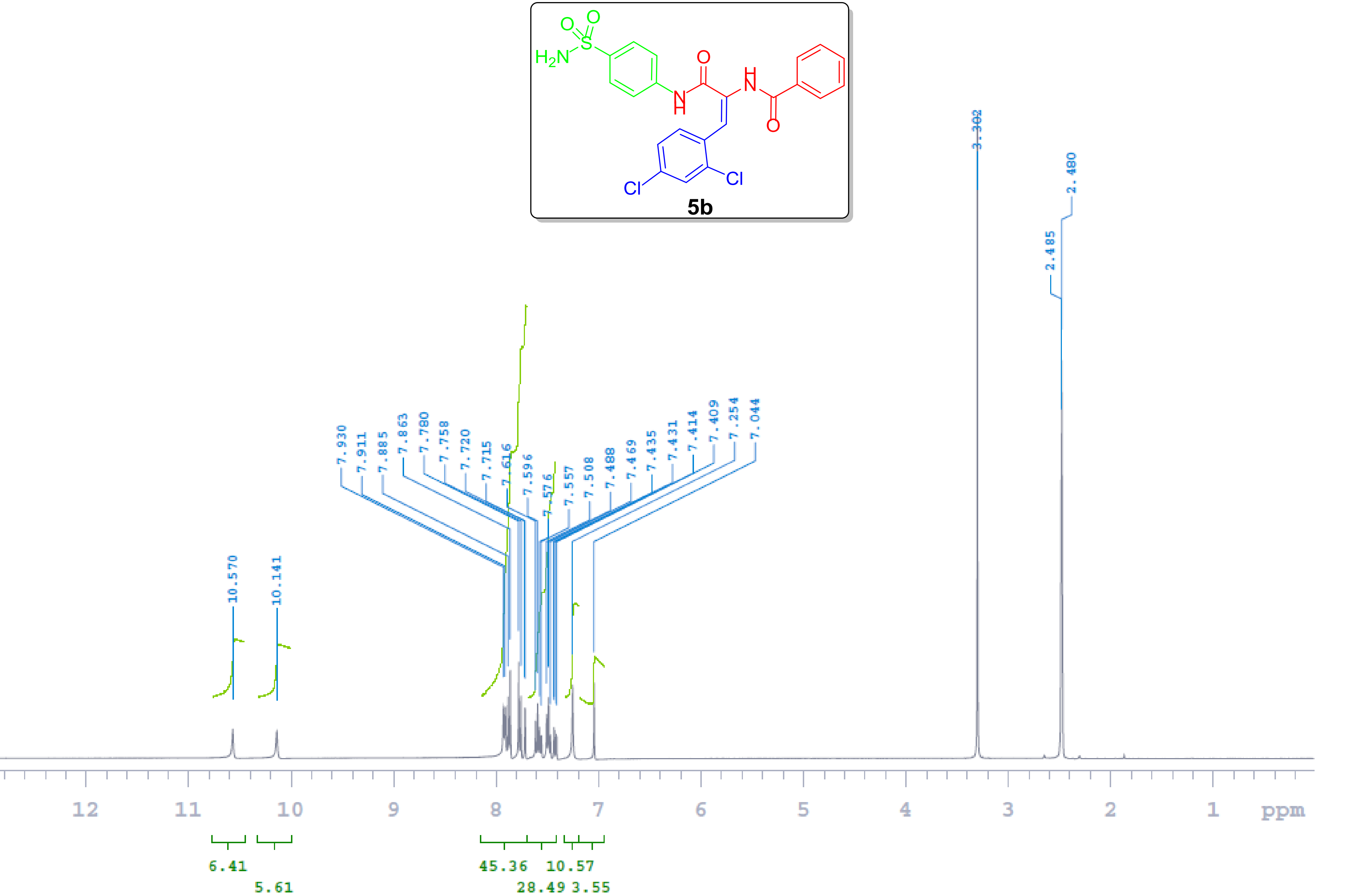

Dr_WagdyMohamed-d-H-D2O

Sample Name	Dr_WagdyMohamed-d-H-D2O	Pulse sequence	PROTON	Temperature	25	Study owner	vnmr1
Date collected	2016-11-20	Solvent	dmso	Spectrometer	nmr400-mercury400	Operator	vnmr1

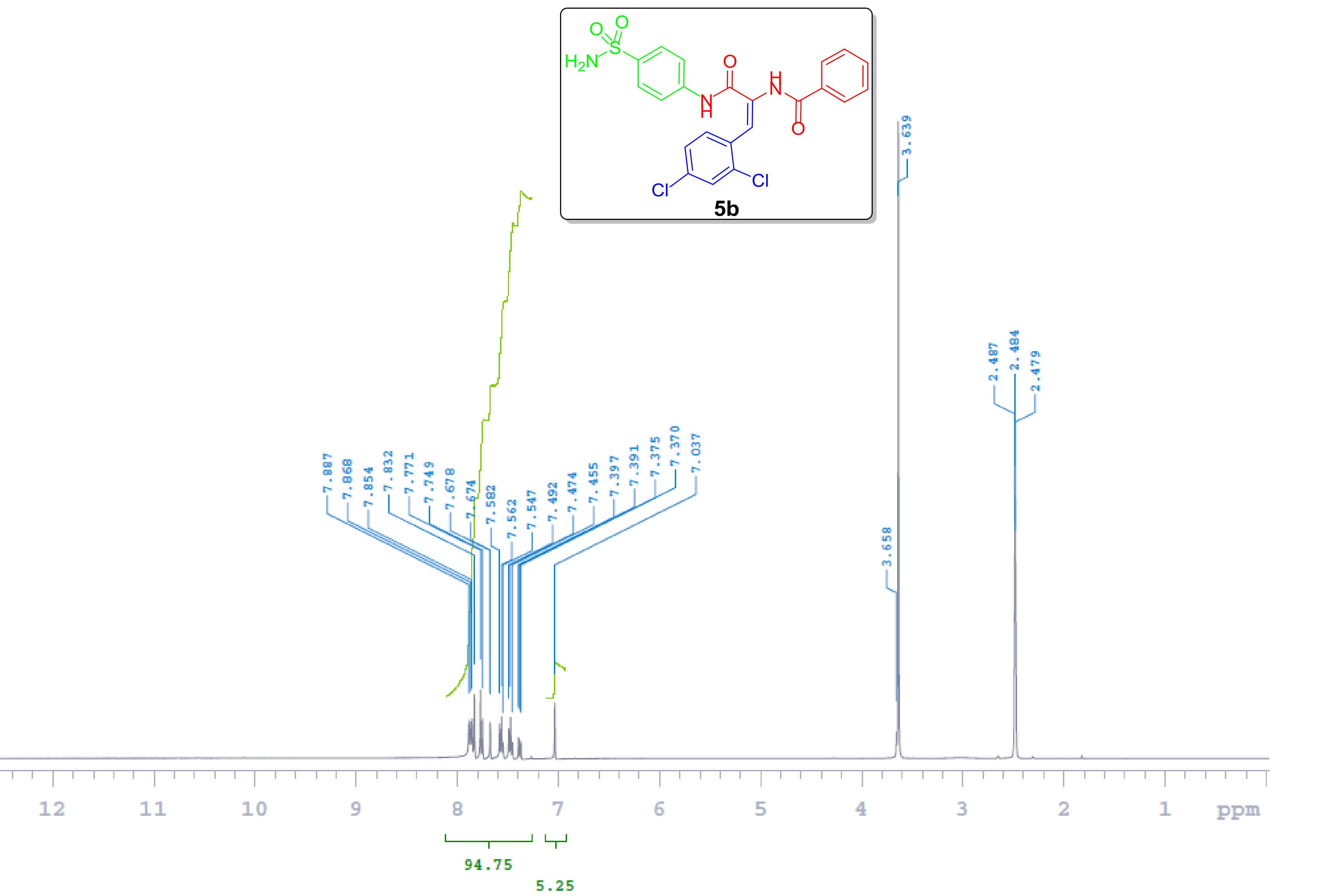

Dr_WagdyMohamed-e_H

Sample Name	Dr_WagdyMohamed-e_H	Pulse sequence	CARBON	Temperature	25	Study owner	vnmr1
Date collected	2017-01-22	Solvent	dmso	Spectrometer	nmr400-mercury400	Operator	vnmr1

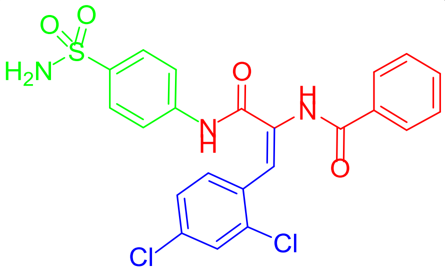

5b

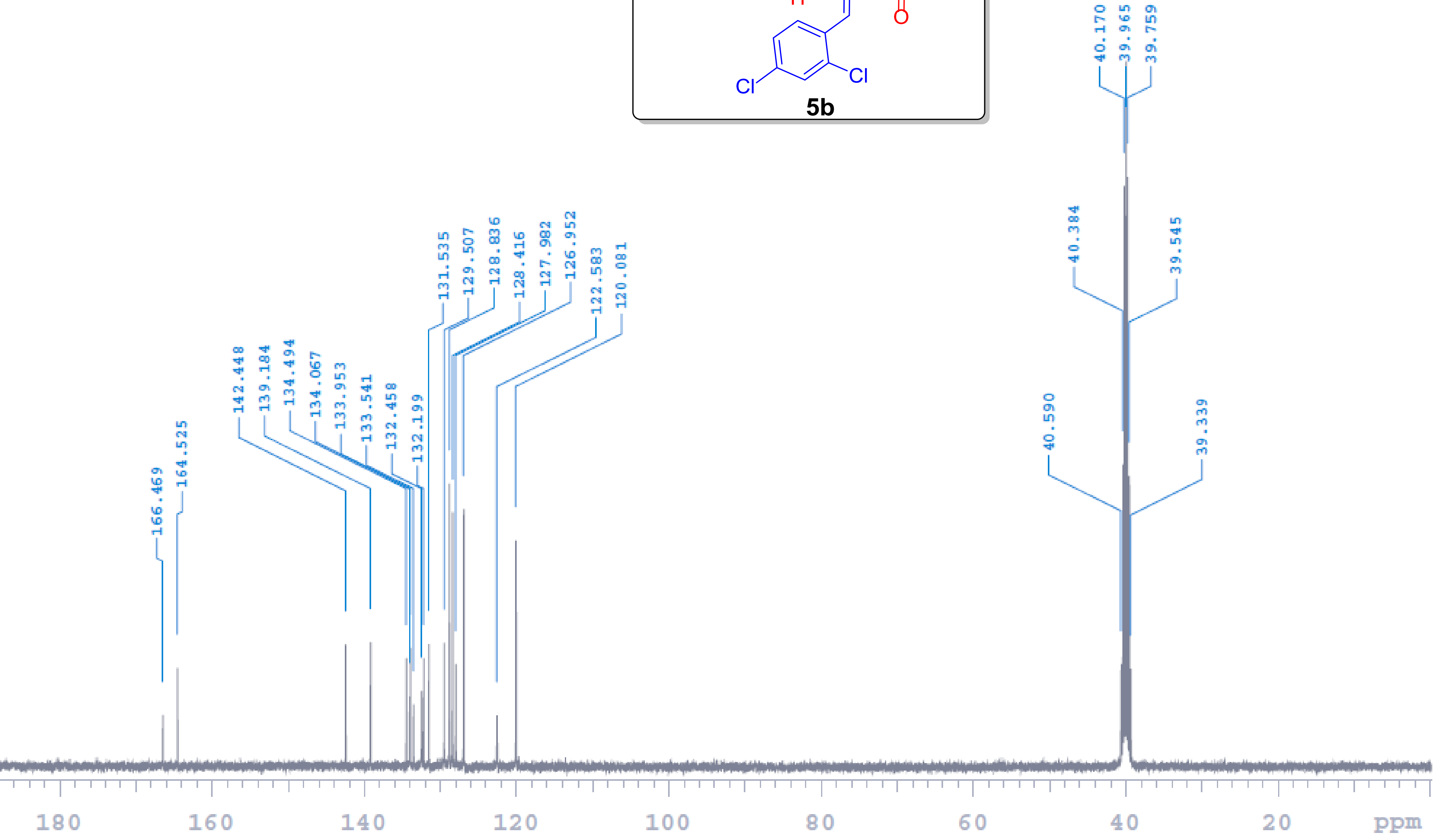

Dr_WagdyMohamed-e-H-D2O

Sample Name	Dr_WagdyMohamed-e-H-D2O	Pulse sequence	PROTON	Temperature	25	Study owner	vnmr1
Date collected	2016-11-20	Solvent	dmso	Spectrometer	nmr400-mercury400	Operator	vnmr1

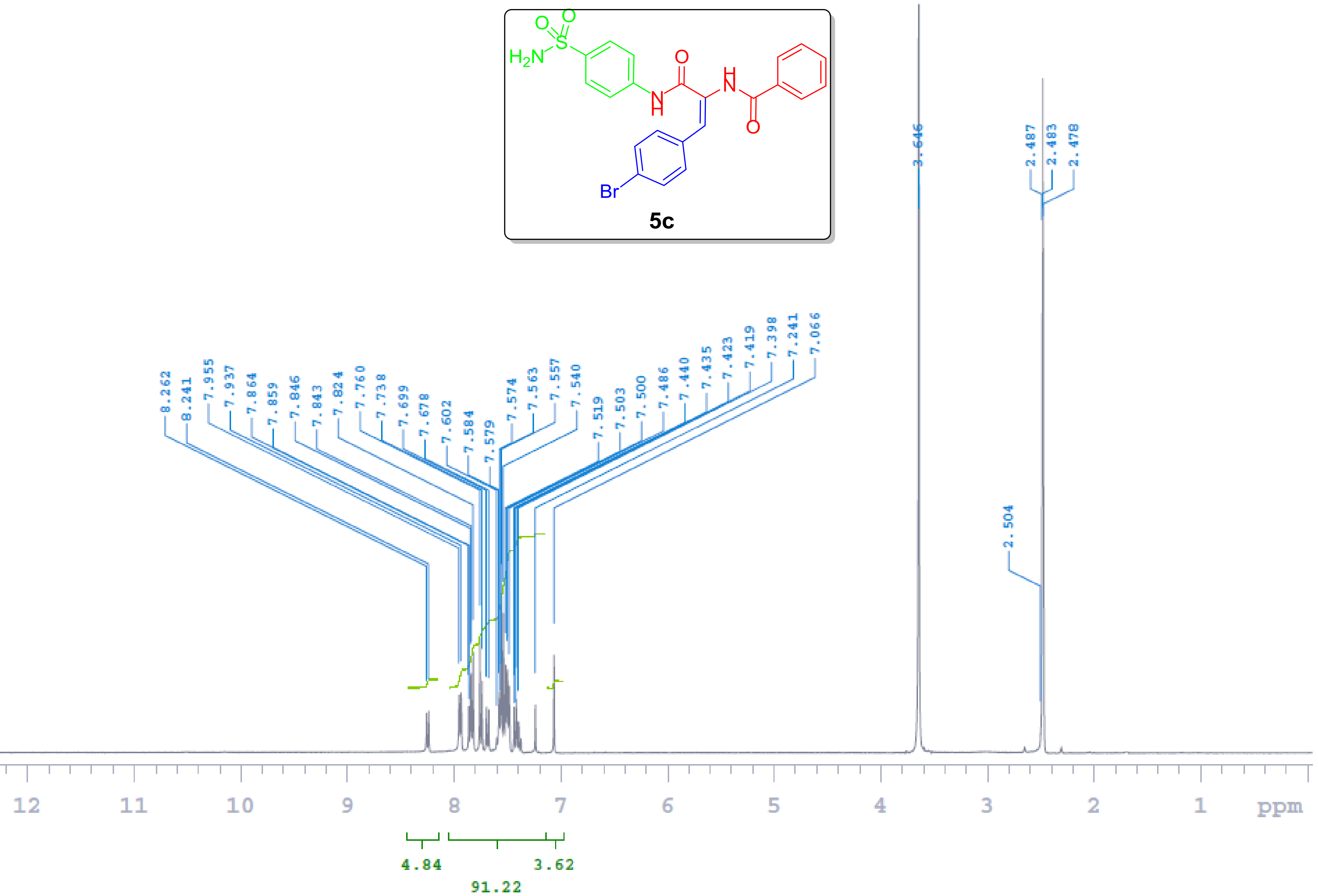

Dr_WagdyMohamed-h_H

Sample Name	Dr_WagdyMohamed-h_H	Pulse sequence	PROTON	Temperature	25	Study owner	vnmr1
Date collected	2017-02-19	Solvent	dmso	Spectrometer	nmr400-mercury400	Operator	vnmr1

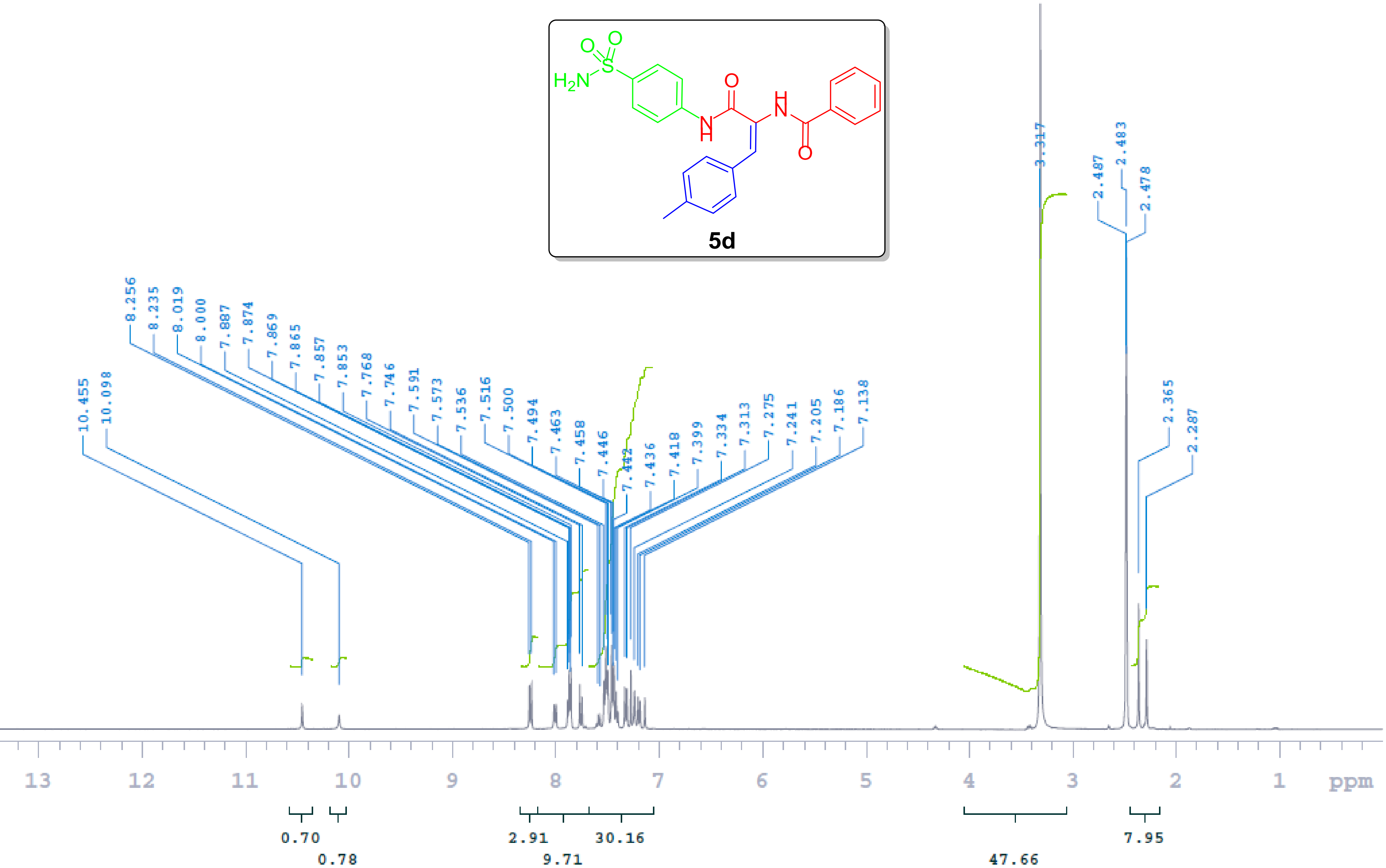

Dr_WagdyMohamed-h_H-D2O

Sample Name	Dr_WagdyMohamed-h_H-D2O	Pulse sequence	PROTON	Temperature	25	Study owner	vnmr1
Date collected	2017-02-20	Solvent	dmso	Spectrometer	nmr400-mercury400	Operator	vnmr1

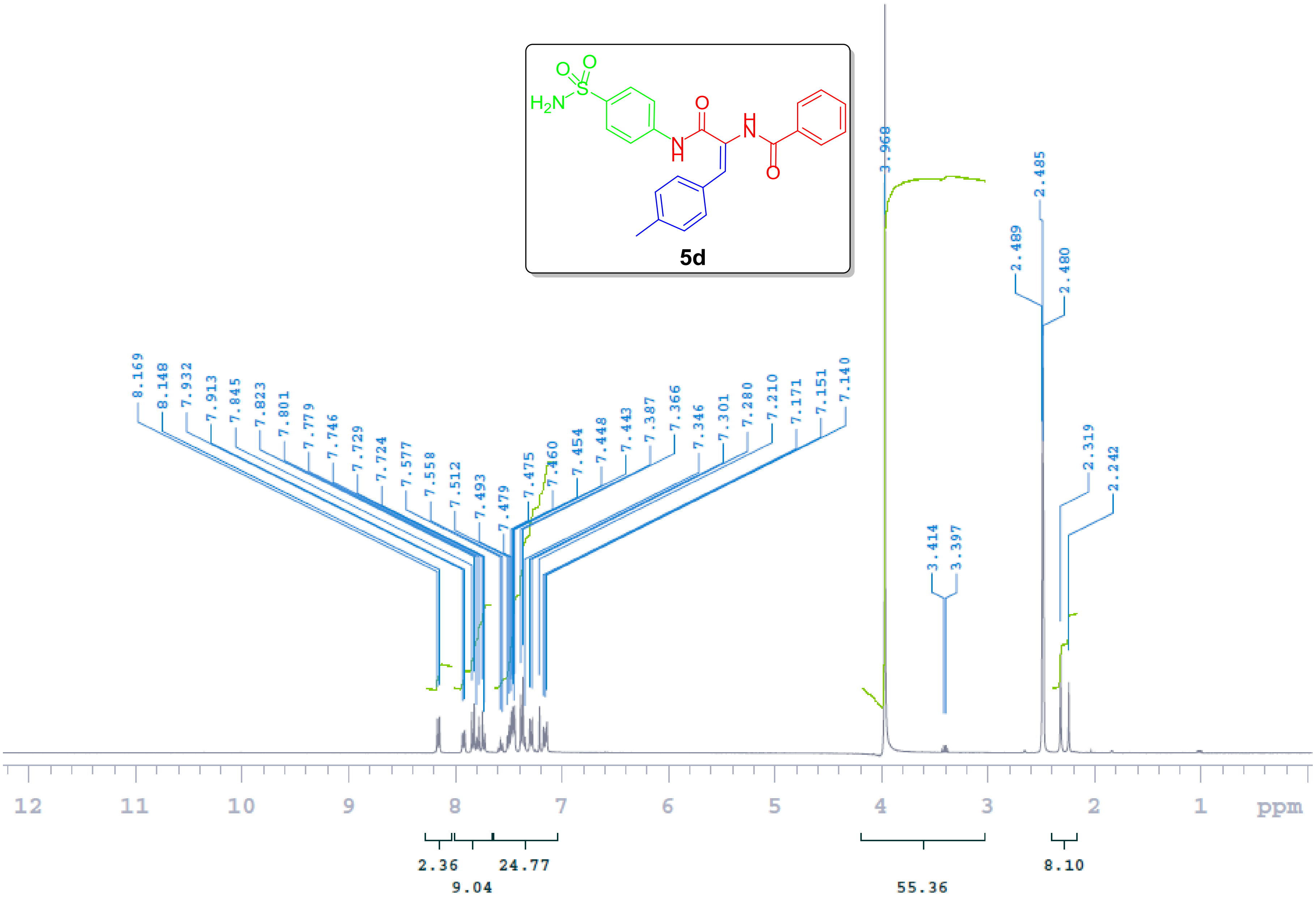

Dr_WagdyMohamed-h_H

Sample Name	Dr_WagdyMohamed-h_H	Pulse sequence	CARBON	Temperature	25	Study owner	vnmr1
Date collected	2017-02-19	Solvent	dmso	Spectrometer	nmr400-mercury400	Operator	vnmr1

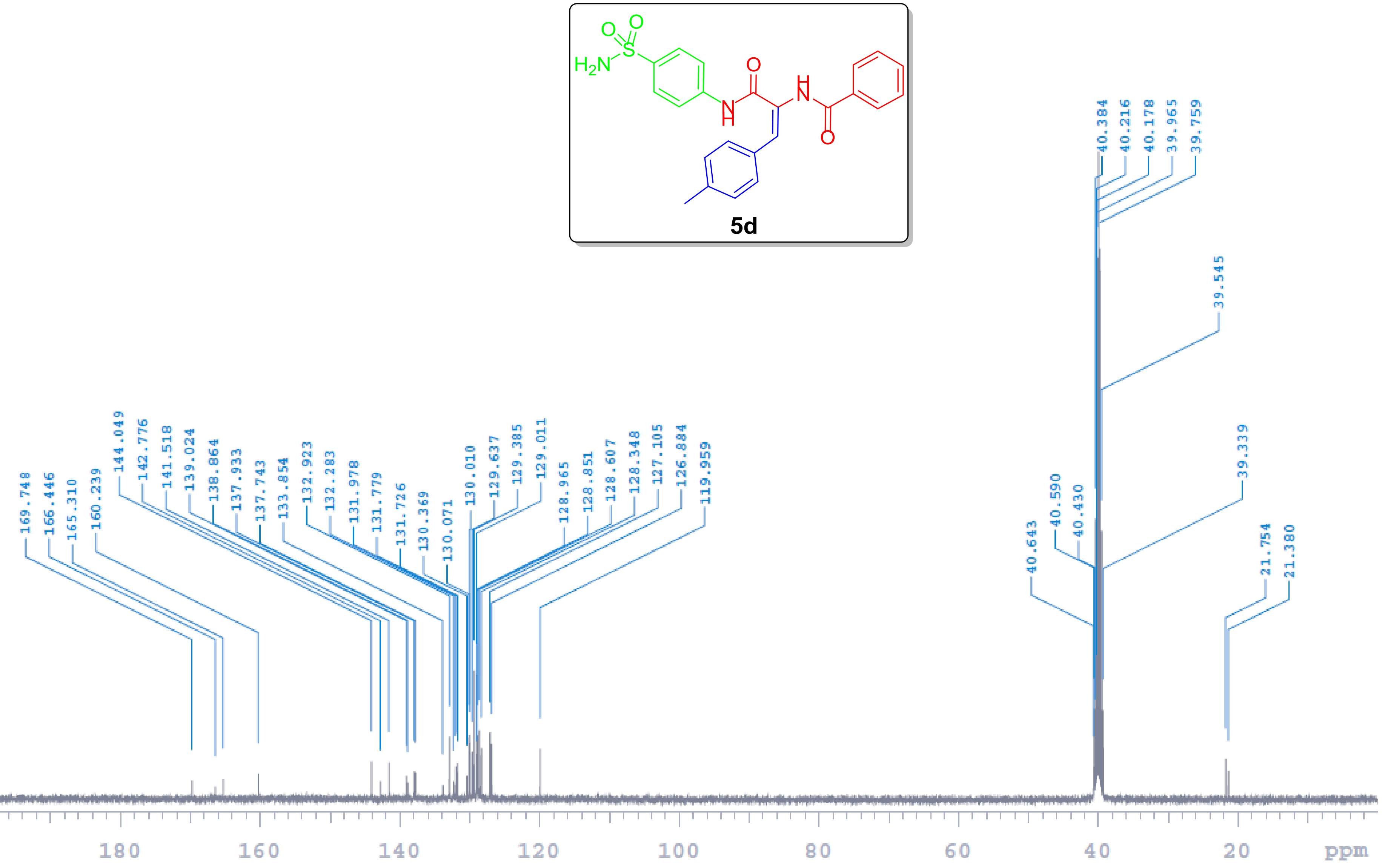

X:\data\Mar 19\ESI75853.raw 27/03/2019 5:04 pm

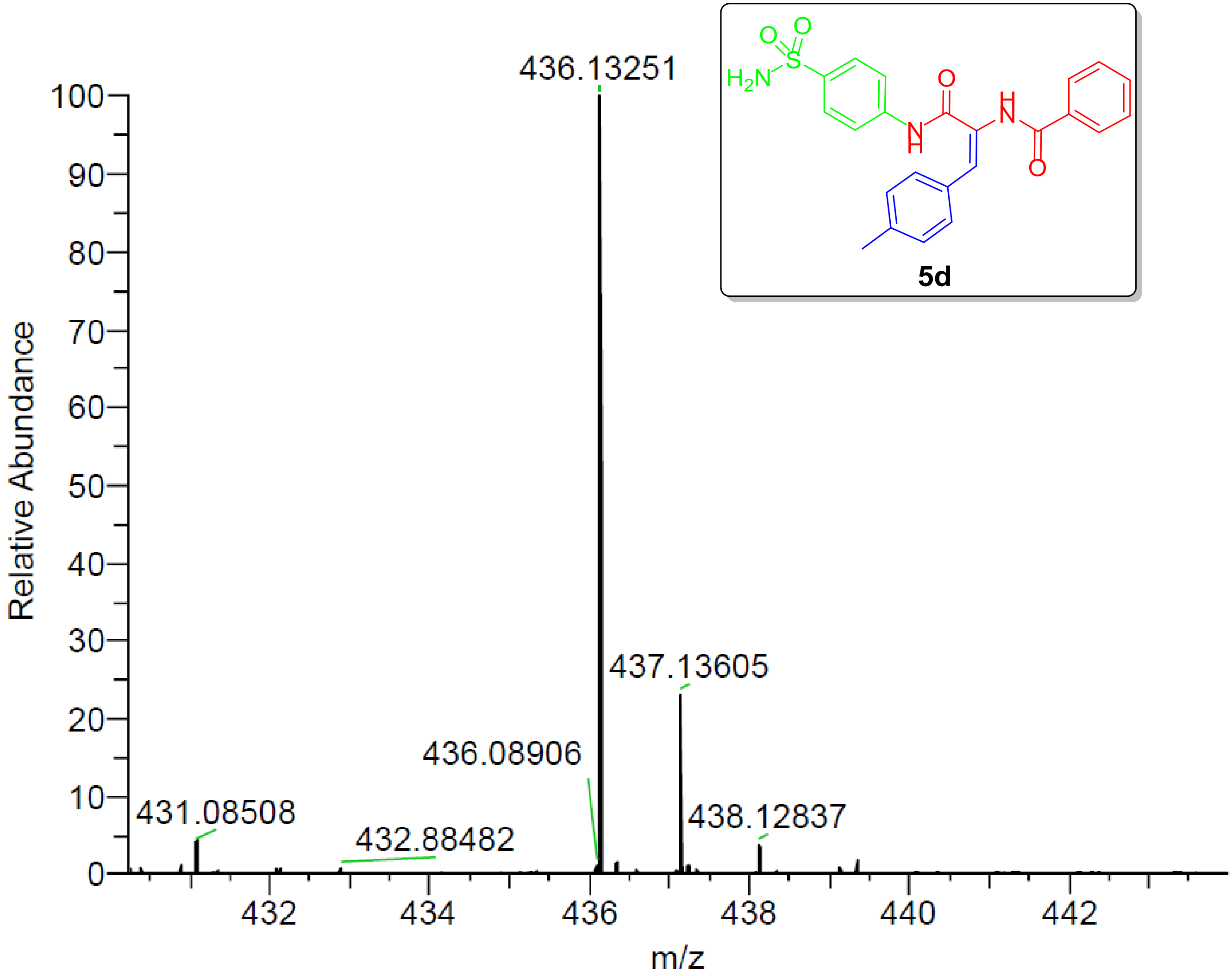

NL: 8.49E5
ESI75853 #13-27 RT: 0.14-0.31 AV: 8 NL: 1.20E+007
T: FTMS {1,1} + p ESI Full lock ms [80.00-1600.00]

Measured Spectrum

Sample Name Dr_WagdyMohamed-i_H-D2O Pulse sequence PROTON Temperature 25 Study owner vnmr1
Date collected 2017-01-24 Solvent dmso Spectrometer nmr400-mercury400 Operator vnmr1

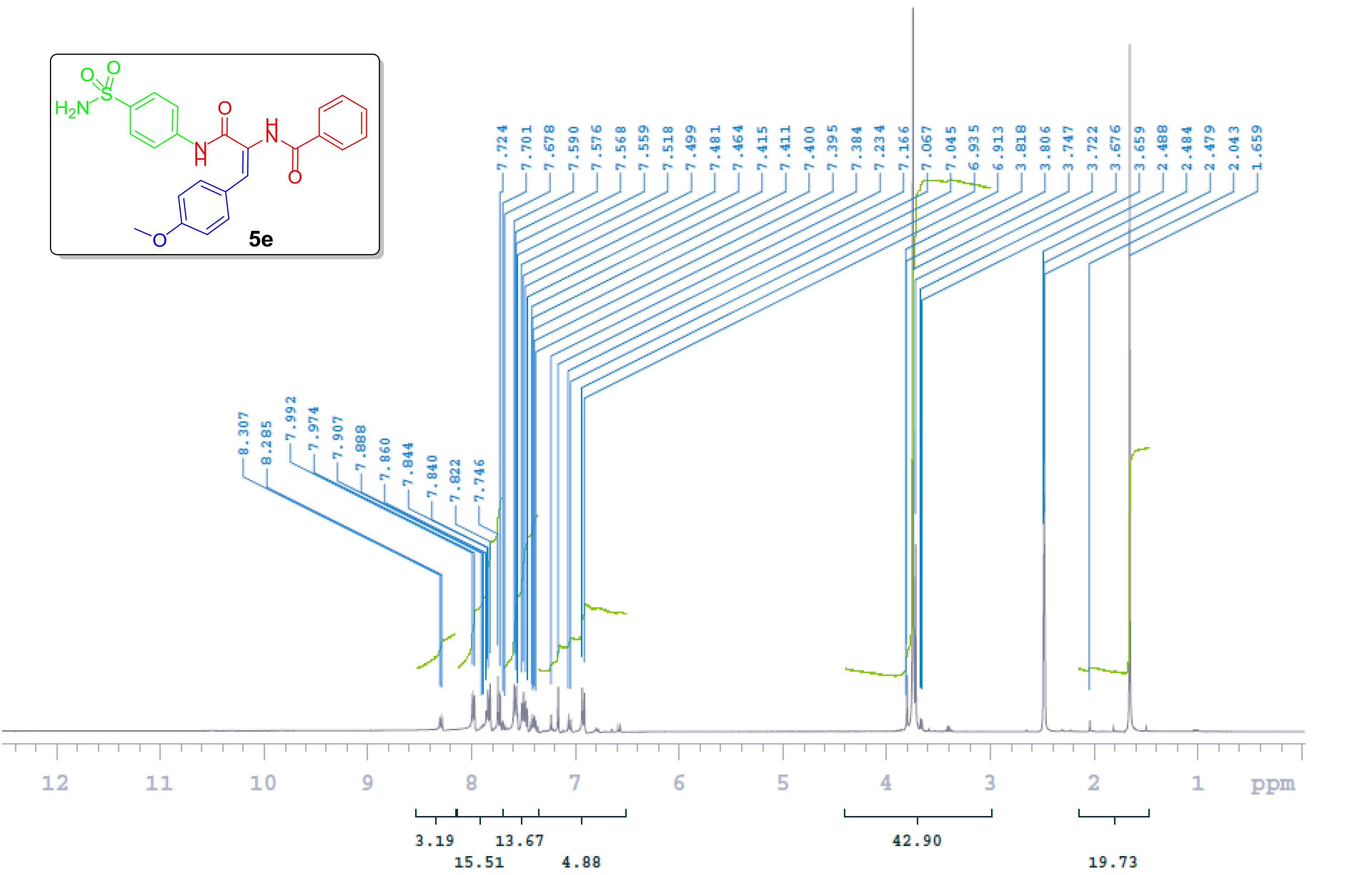

Dr_WagdyMohamed-i_H

Sample Name	Dr_WagdyMohamed-i_H	Pulse sequence	PROTON	Temperature	25	Study owner	vnmr1
Date collected	2017-01-23	Solvent	dmso	Spectrometer	nmr400-mercury400	Operator	vnmr1

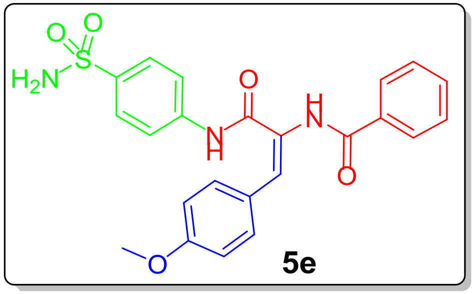

5e

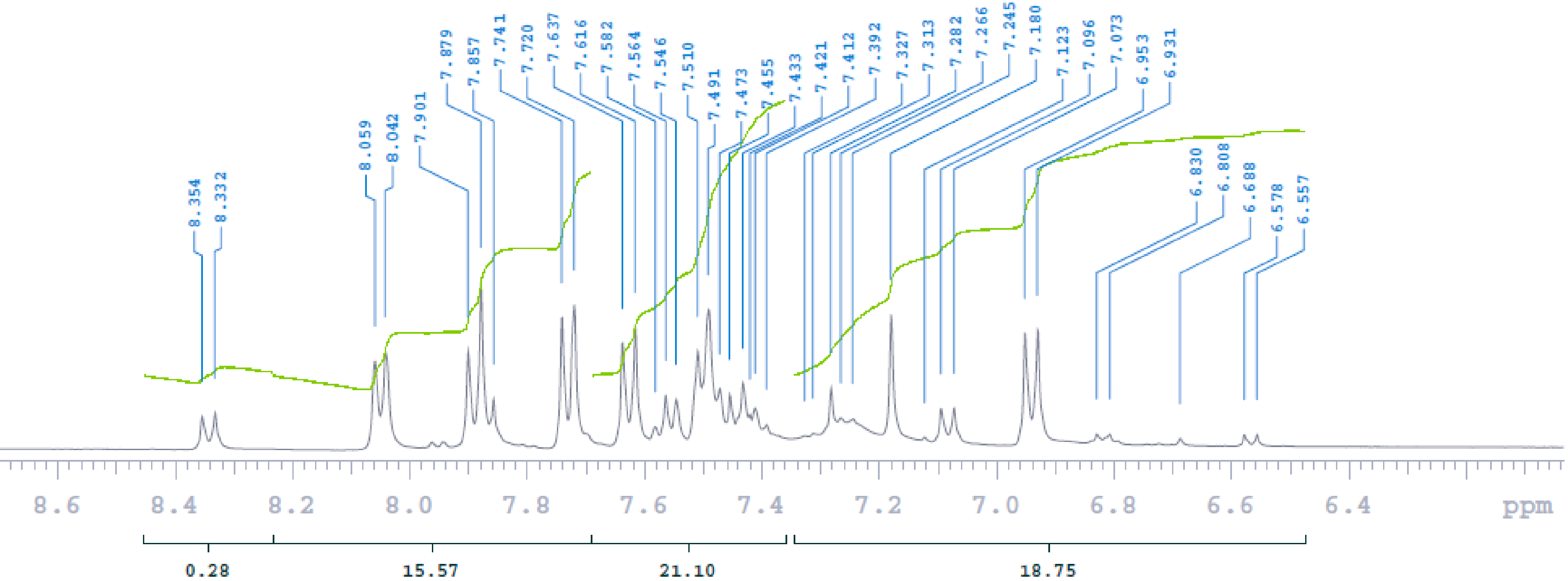

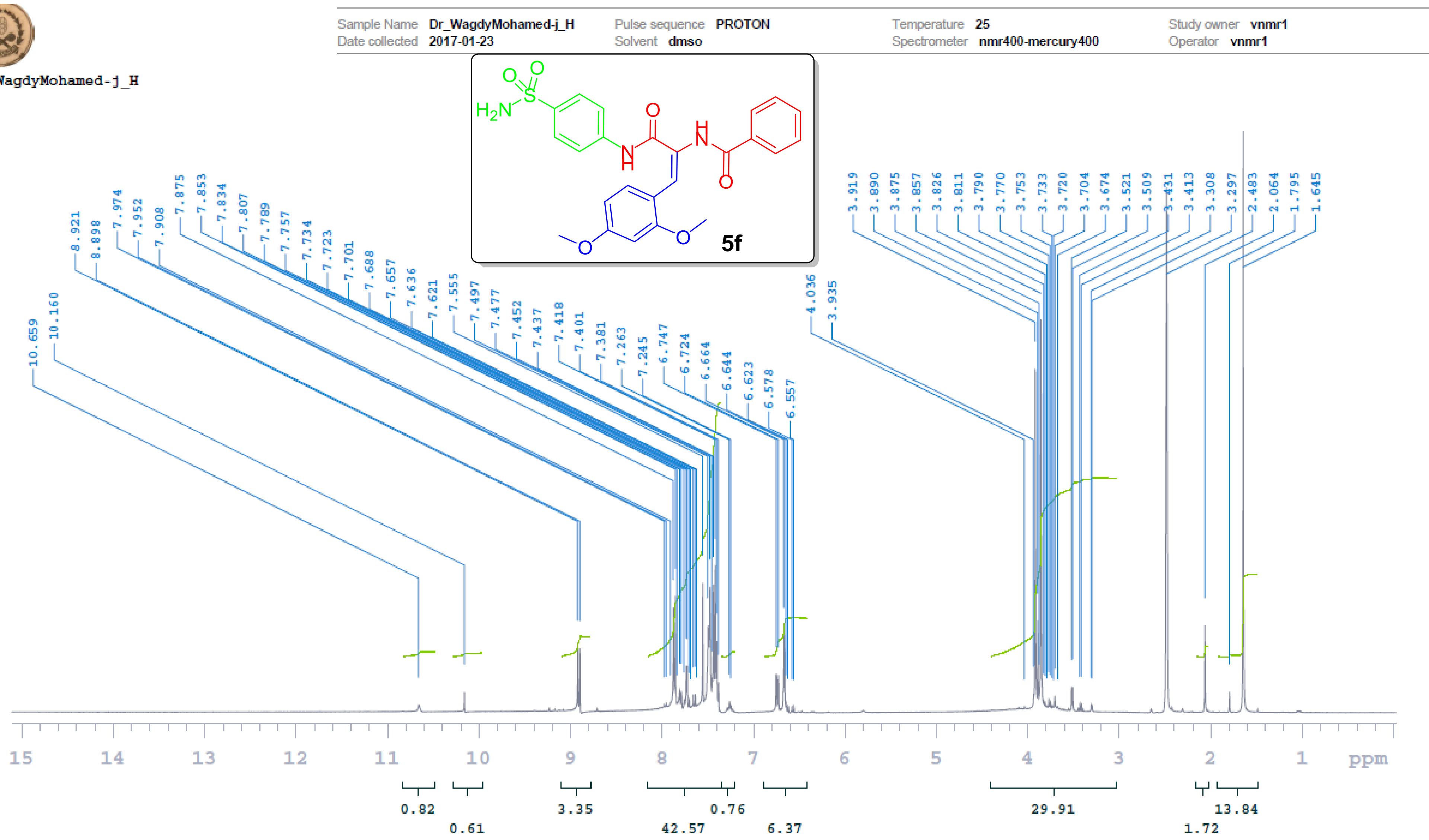

Sample Name Dr_WagdyMohamed-j_H
Date collected 2017-01-23
Pulse sequence PROTON
Solvent dmso
Temperature 25
Spectrometer nmr400-mercury400
Study owner vnmr1
Operator vnmr1
WagdyMohamed-j_H
H_2N
S
O
O
N
H
O
N
H
O
O
O
5f
10.659
10.160
8.921
8.898
7.974
7.952
7.908
7.875
7.853
7.834
7.807
7.789
7.757
7.734
7.723
7.701
7.688
7.657
7.636
7.621
7.555
7.497
7.477
7.452
7.437
7.418
7.401
7.381
7.263
7.245
6.747
6.724
6.664
6.644
6.623
6.578
6.557
4.036
3.935
3.919
3.890
3.875
3.857
3.826
3.811
3.790
3.770
3.753
3.733
3.720
3.704
3.674
3.521
3.509
3.431
3.413
3.308
3.297
2.483
2.064
1.795
1.645
15
14
13
12
11
10
9
8
7
6
5
4
3
2
1
ppm
0.82
0.61
3.35
42.57
0.76
6.37
29.91
1.72
13.84

Dr_WagdyMohamed-k_H

Sample Name Dr_WagdyMohamed-k_H | Pulse sequence PROTON | Temperature 25 | Study owner vnmr1

Date collected 2017-02-19 | Solvent dmso | Spectrometer nmr400-mercury400 | Operator vnmr1

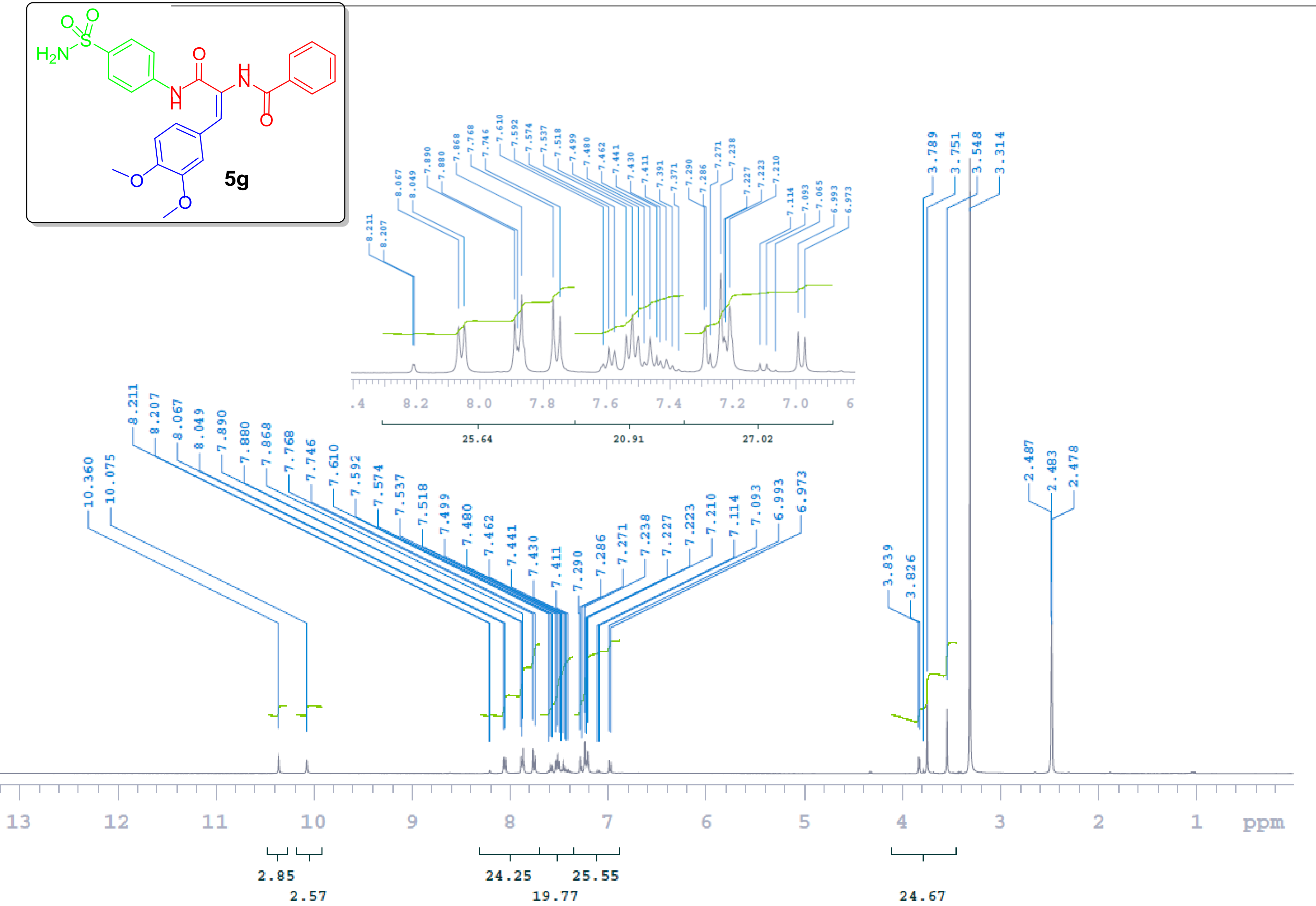

Dr_WagdyMohamed-K_H-D2O

Sample Name Dr_WagdyMohamed-K_H-D2O Pulse sequence PROTON Temperature 25 Study owner vnmr1
Date collected 2017-02-20 Solvent dmso Spectrometer nmr400-mercury400 Operator vnmr1

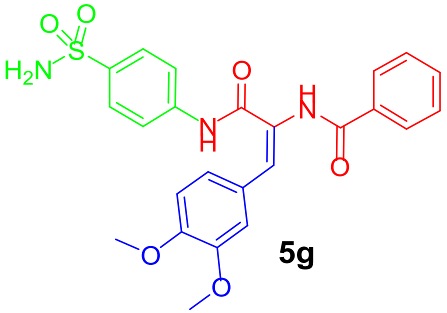

5g

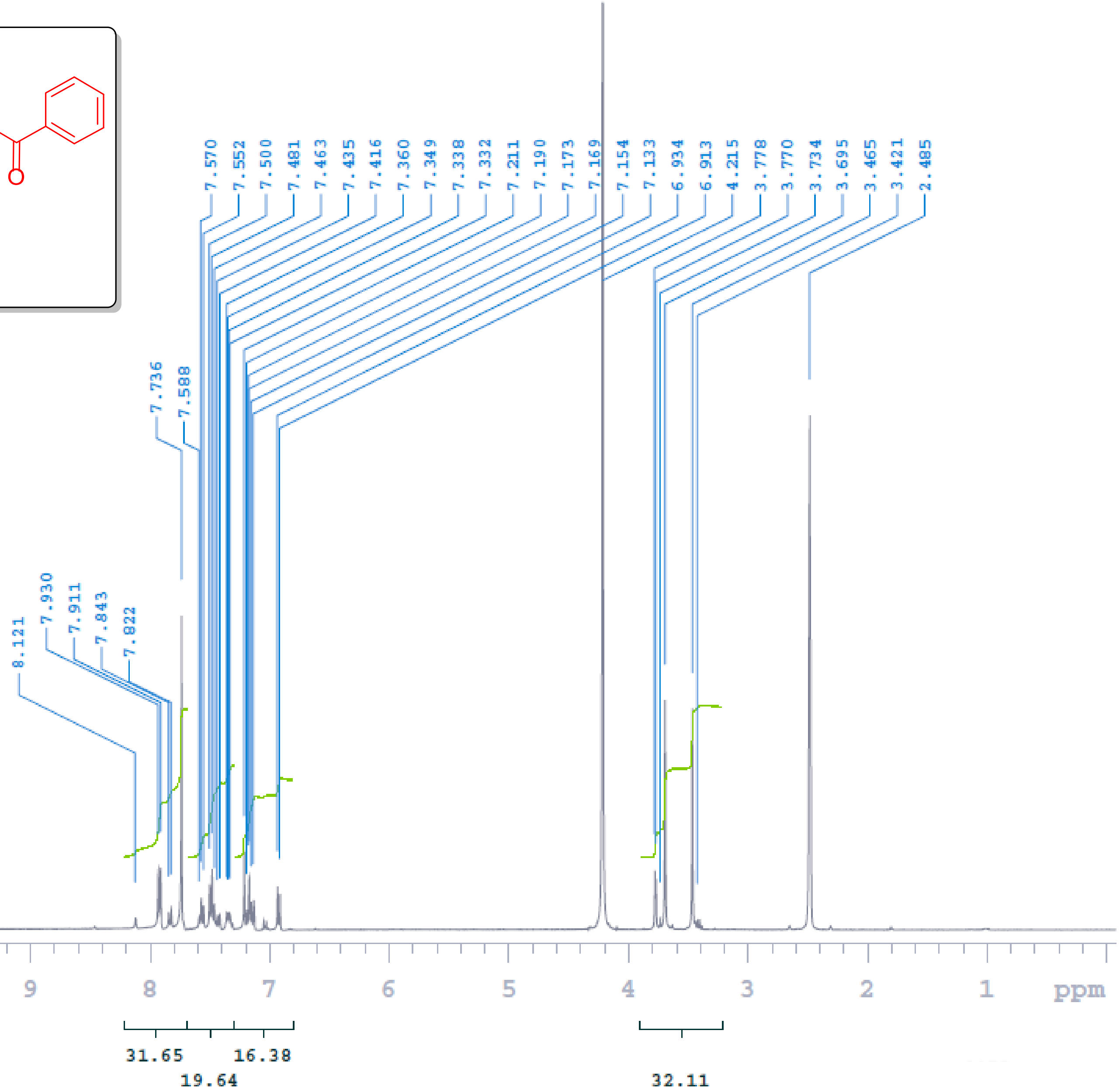

Dr_WagdyMohamed-k_H

Sample Name	Dr_WagdyMohamed-k_H	Pulse sequence	CARBON	Temperature	25	Study owner	vnmr1
Date collected	2017-02-19	Solvent	dmso	Spectrometer	nmr400-mercury400	Operator	vnmr1

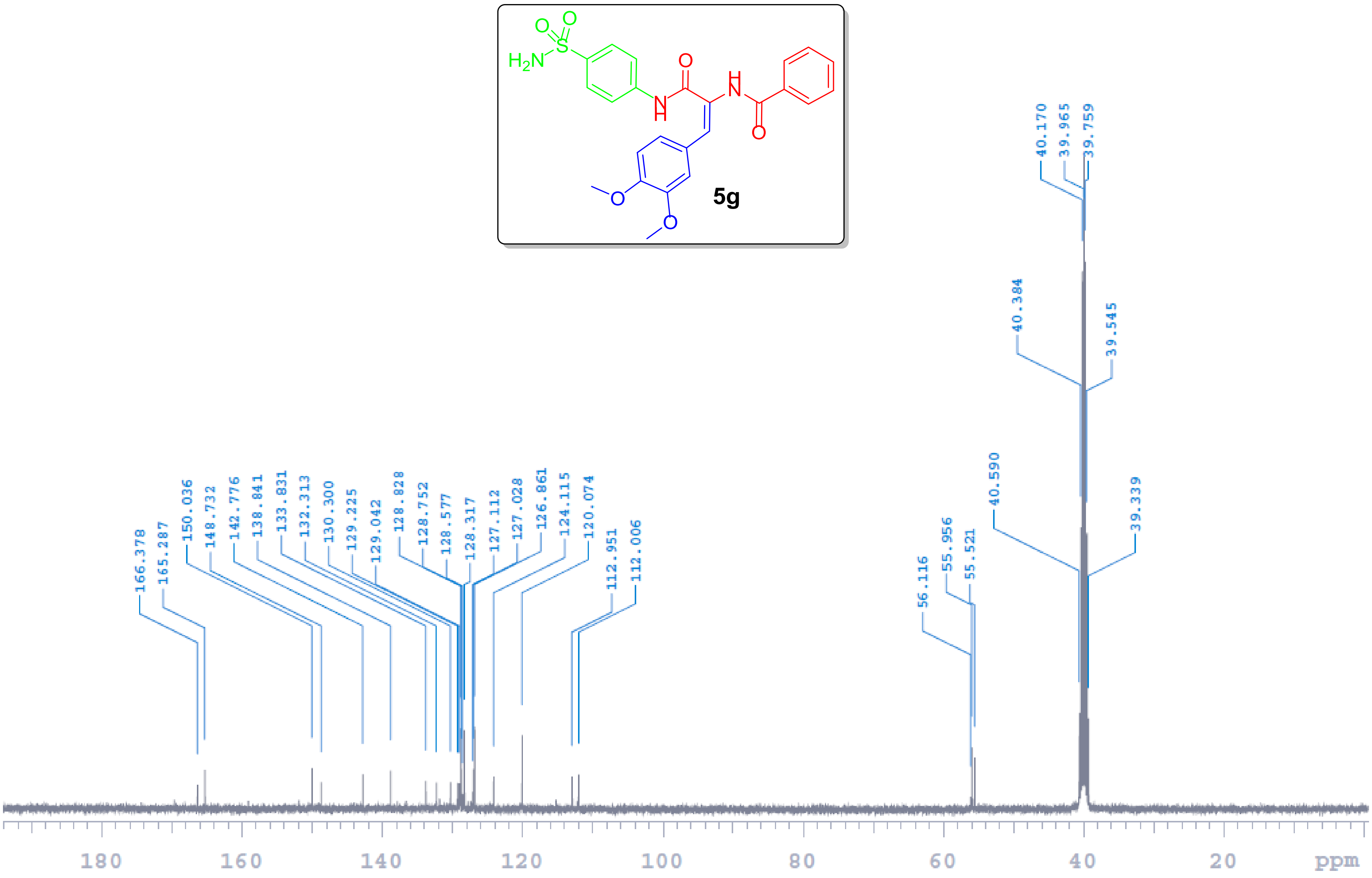

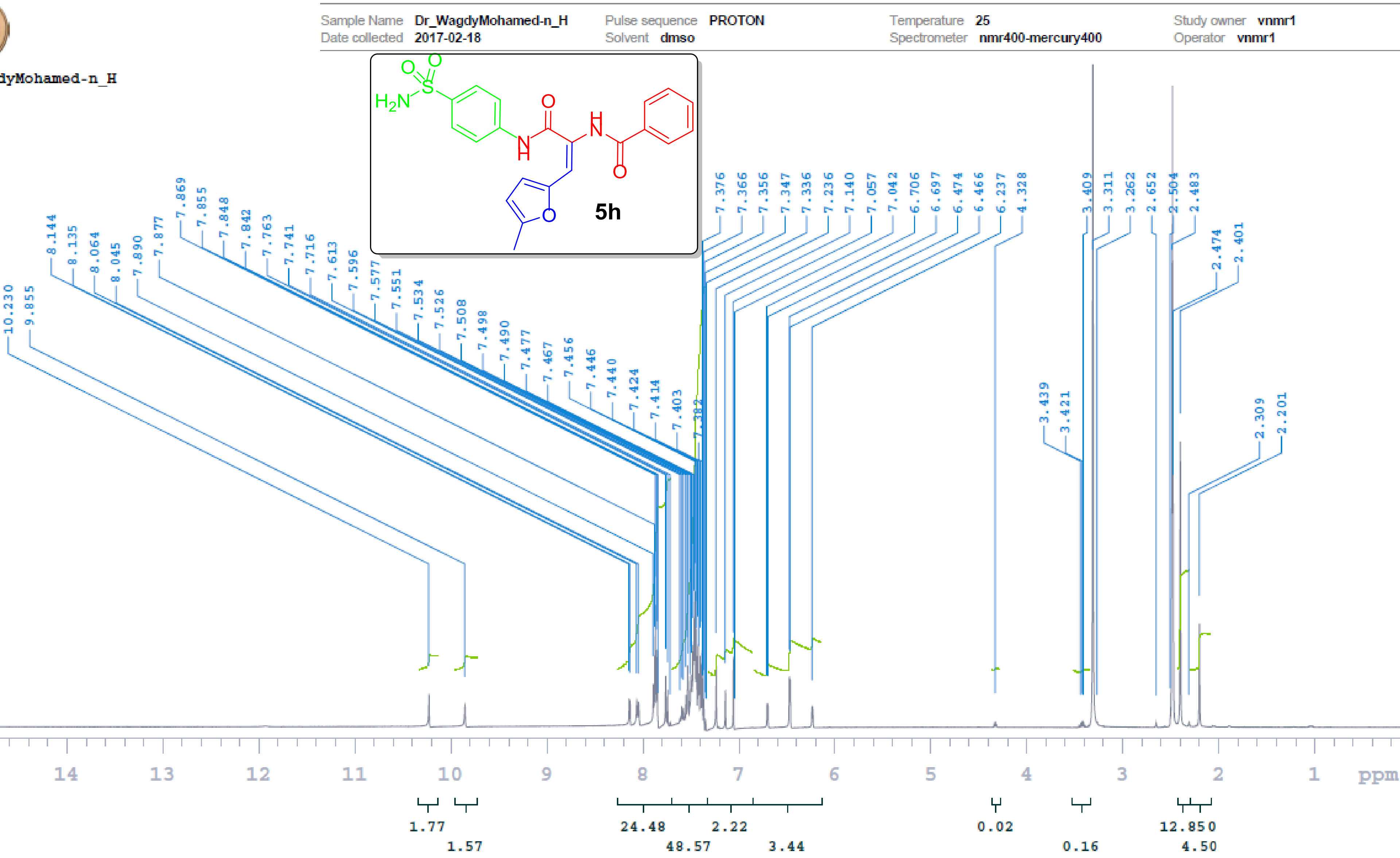
dyMohamed-n_H
Sample Name Dr_WagdyMohamed-n_H
Date collected 2017-02-18
Pulse sequence PROTON
Solvent dmso
Temperature 25
Spectrometer nmr400-mercury400
Study owner vnmr1
Operator vnmr1
5h
10.230 9.855 8.144 8.135 8.064 8.045 7.890 7.877 7.869 7.855 7.848 7.842 7.763 7.741 7.716 7.613 7.596 7.577 7.551 7.534 7.526 7.508 7.498 7.490 7.477 7.467 7.456 7.446 7.440 7.424 7.414 7.403 7.382
7.376 7.366 7.356 7.347 7.336 7.236 7.140 7.057 7.042 6.706 6.697 6.474 6.466 6.237 4.328
3.439 3.421 3.409 3.311 3.262 2.652 2.504 2.483 2.474 2.401 2.309 2.201
14 13 12 11 10 9 8 7 6 5 4 3 2 1 ppm
1.77 1.57 24.48 48.57 2.22 3.44 0.02 0.16 12.850 4.50

Dr_WagdyMohamed-n_H

Dr_WagdyMohamed-n_H

Sample Name	Dr_WagdyMohamed-n_H	Pulse sequence	CARBON	Temperature	25	Study owner	vnmr1
Date collected	2017-02-18	Solvent	dmso	Spectrometer	nmr400-mercury400	Operator	vnmr1

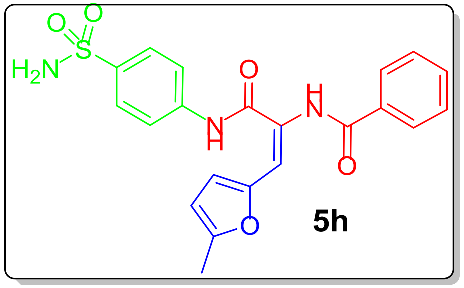

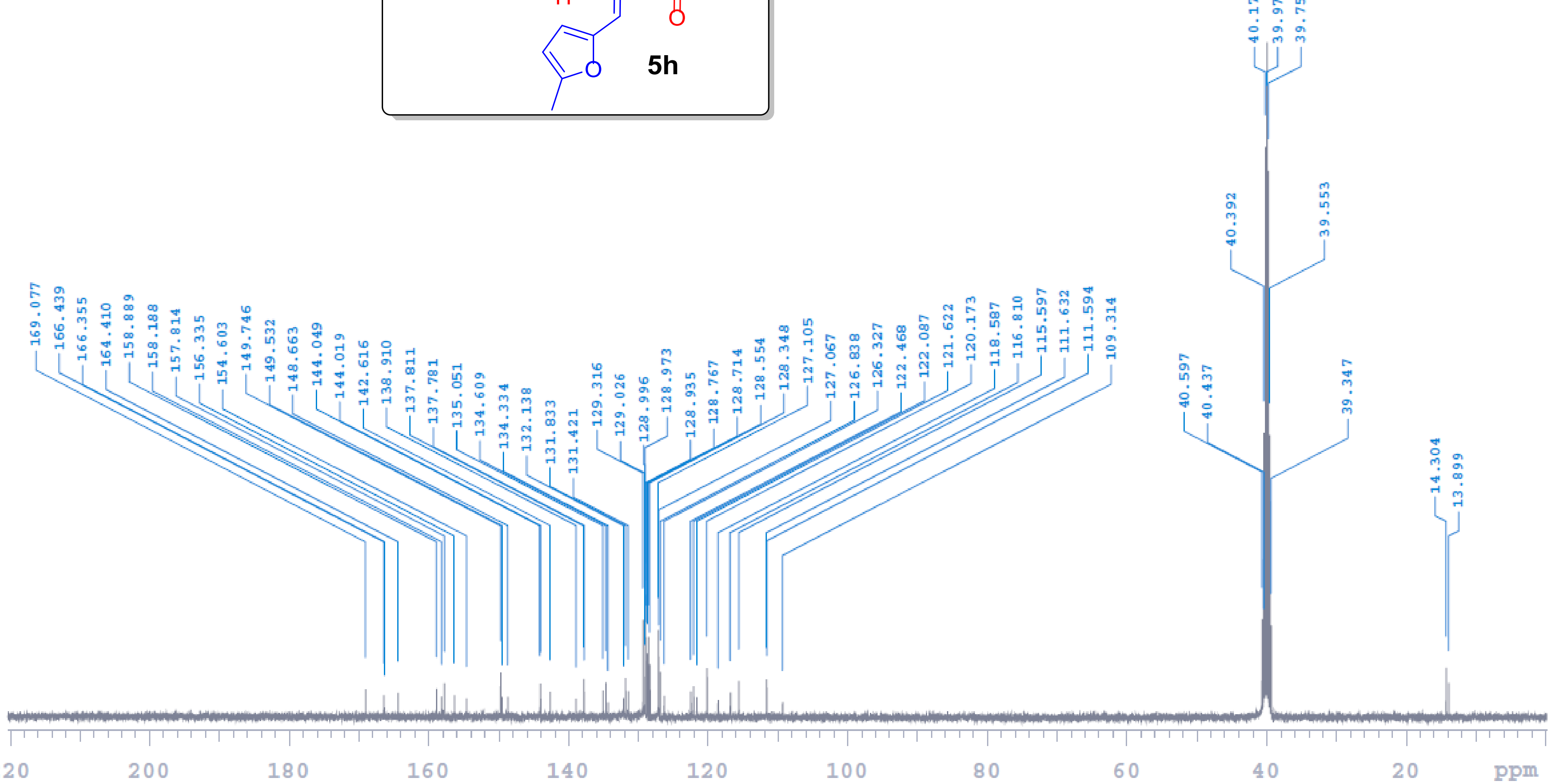

Developmental Therapeutics Program

One Dose Mean Graph 5a

NSC: D-808740 / 1	Conc: 1.00E-5 Molar	Test Date: Oct 01, 2018
Experiment ID: 1810OS18		Report Date: Oct 28, 2018

Panel/Cell Line	Growth Percent
**Leukemia**	
CCRF-CEM	107.14
HL-60(TB)	101.21
K-562	109.37
MOLT-4	102.83
RPMI-8226	109.30
SR	96.44
**Non-Small Cell Lung Cancer**	
A549/ATCC	101.38
EKVX	99.55
HOP-62	91.94
HOP-92	98.77
NCI-H226	92.90
NCI-H23	102.62
NCI-H322M	107.87
NCI-H460	105.66
NCI-H522	90.90
**Colon Cancer**	
COLO 205	104.19
HCC-2998	102.74
HCT-116	93.94
HCT-15	105.78
HT29	105.51
KM12	108.06
SW-620	100.29
**CNS Cancer**	
SF-268	95.99
SF-295	104.10
SF-539	98.82
SNB-19	102.93
SNB-75	86.28
U251	95.34
**Melanoma**	
LOX IMVI	97.02
MALME-3M	96.29
M14	102.35
MDA-MB-435	98.69
SK-MEL-2	102.02
SK-MEL-28	109.96
SK-MEL-5	100.03
UACC-257	102.61
UACC-62	96.17
**Ovarian Cancer**	
IGROV1	90.87
OVCAR-3	104.30
OVCAR-4	95.47
OVCAR-5	104.86
OVCAR-8	97.63
NCI/ADR-RES	104.11
SK-OV-3	97.03
**Renal Cancer**	
786-0	95.86
A498	103.52
ACHN	100.19
CAKI-1	92.48
RXF 393	102.78
SN12C	103.74
TK-10	96.59
UO-31	78.68
**Prostate Cancer**	
PC-3	104.83
DU-145	107.47
**Breast Cancer**	
MCF7	98.34
MDA-MB-231/ATCC	105.37
HS 578T	101.13
T-47D	95.50
MDA-MB-468	100.89
Mean	100.15
Delta	21.47
Range	31.28

Mean Growth Percent - Growth Percent

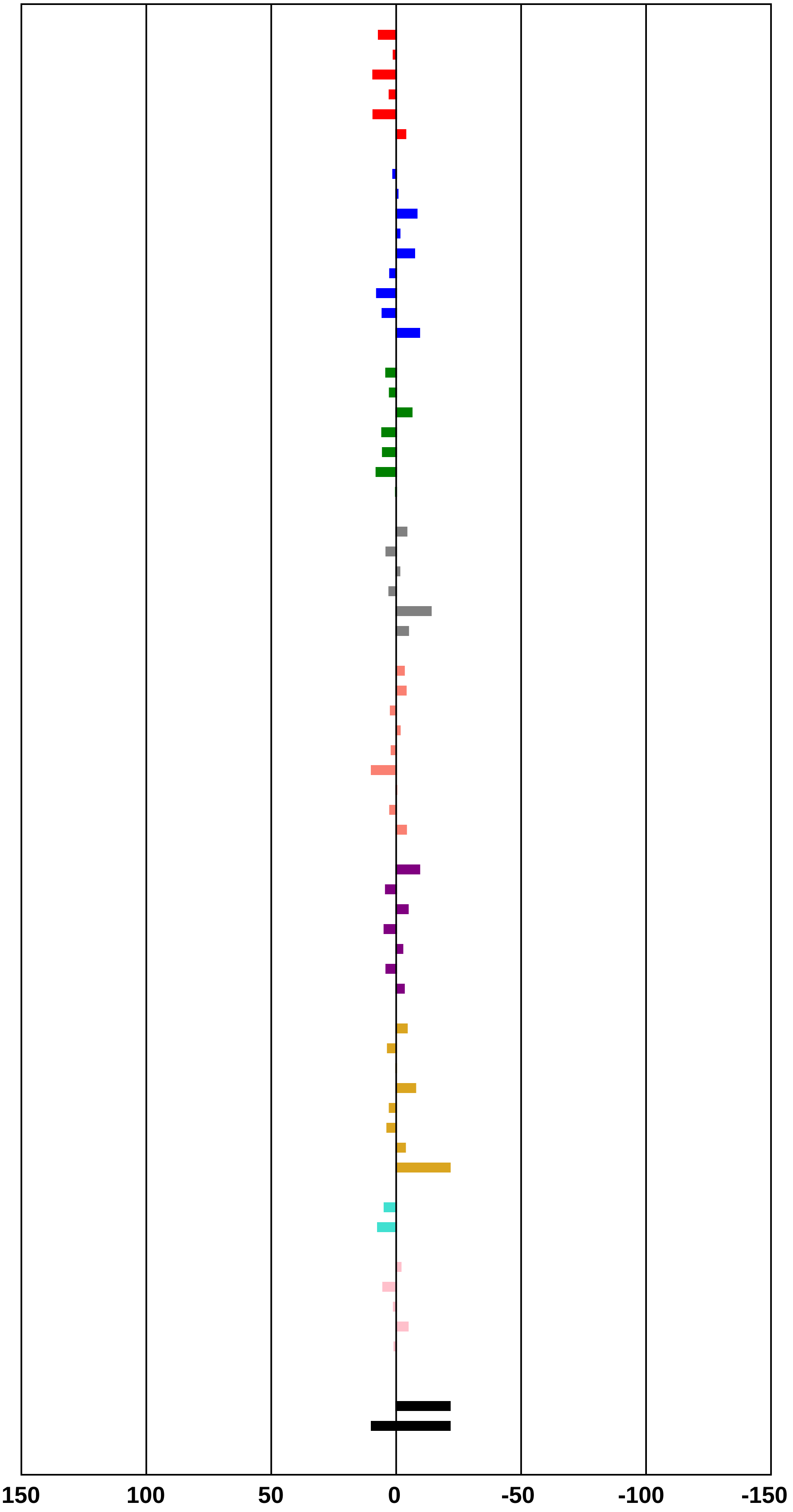

Developmental Therapeutics Program

One Dose Mean Graph 5b

NSC: D-808667 / 1	Conc: 1.00E-5 Molar	Test Date: Oct 01, 2018
Experiment ID: 1810OS18		Report Date: Oct 28, 2018

Panel/Cell Line	Growth Percent
**Leukemia**	
CCRF-CEM	104.89
HL-60(TB)	84.77
K-562	88.21
MOLT-4	92.28
RPMI-8226	92.16
SR	96.62
**Non-Small Cell Lung Cancer**	
A549/ATCC	104.81
EKVX	99.61
HOP-62	95.67
HOP-92	87.01
NCI-H226	102.97
NCI-H23	106.78
NCI-H322M	102.44
NCI-H460	104.96
NCI-H522	80.34
**Colon Cancer**	
COLO 205	110.30
HCC-2998	103.39
HCT-116	97.28
HCT-15	110.25
HT29	102.93
KM12	102.43
SW-620	103.87
**CNS Cancer**	
SF-268	94.50
SF-295	104.17
SF-539	95.74
SNB-19	95.64
SNB-75	81.43
U251	97.14
**Melanoma**	
LOX IMVI	96.20
MALME-3M	93.20
M14	102.47
MDA-MB-435	97.73
SK-MEL-2	95.34
SK-MEL-28	110.07
SK-MEL-5	90.18
UACC-257	101.79
UACC-62	74.95
**Ovarian Cancer**	
IGROV1	74.89
OVCAR-3	112.96
OVCAR-4	97.74
OVCAR-5	107.74
OVCAR-8	100.65
NCI/ADR-RES	98.23
SK-OV-3	96.27
**Renal Cancer**	
786-0	103.70
A498	95.05
ACHN	98.18
CAKI-1	88.64
RXF 393	96.04
SN12C	94.69
TK-10	104.34
UO-31	75.76
**Prostate Cancer**	
PC-3	89.40
DU-145	108.64
**Breast Cancer**	
MCF7	90.11
MDA-MB-231/ATCC	105.22
HS 578T	89.55
BT-549	89.13
T-47D	87.53
MDA-MB-468	110.20
Mean	96.99
Delta	22.10
Range	38.07

Mean Growth Percent - Growth Percent

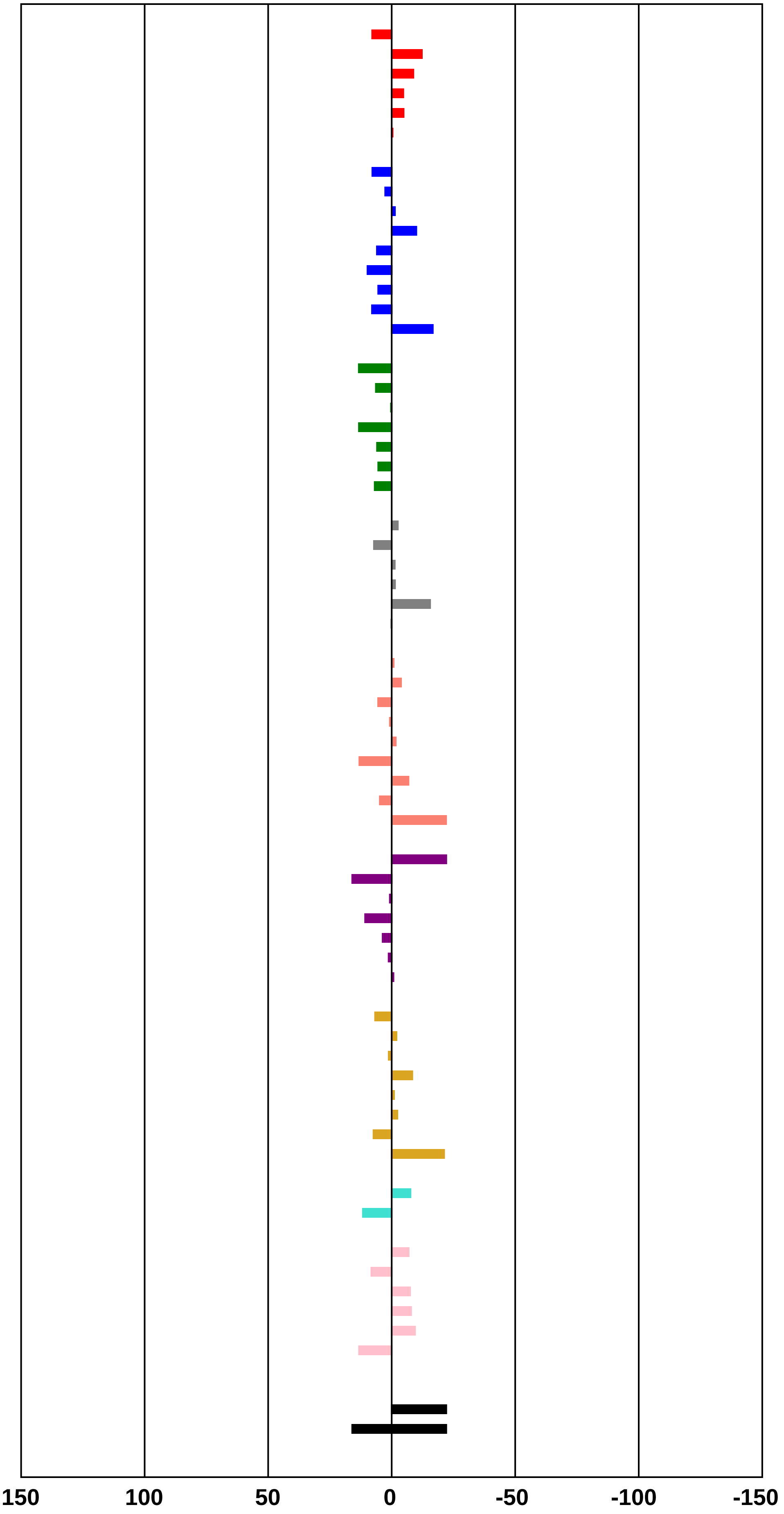

Developmental Therapeutics Program	NSC: D-808669 / 1	Conc: 1.00E-5 Molar	Test Date: Oct 01, 2018
One Dose Mean Graph 5c	Experiment ID: 1810OS18		Report Date: Oct 28, 2018

Panel/Cell Line	Growth Percent
Leukemia	
CCRF-CEM	99.44
HL-60(TB)	96.87
K-562	92.82
MOLT-4	89.47
RPMI-8226	97.16
SR	88.11
Non-Small Cell Lung Cancer	
A549/ATCC	101.70
EKVX	102.00
HOP-62	104.27
HOP-92	93.98
NCI-H226	97.21
NCI-H23	100.45
NCI-H322M	100.50
NCI-H460	106.85
NCI-H522	87.86
Colon Cancer	
COLO 205	104.57
HCC-2998	103.15
HCT-116	92.03
HCT-15	92.53
HT29	89.14
KM12	101.62
SW-620	103.57
CNS Cancer	
SF-268	99.60
SF-295	99.18
SF-539	92.75
SNB-19	95.10
SNB-75	86.46
U251	93.13
Melanoma	
LOX IMVI	99.69
MALME-3M	92.16
M14	109.07
MDA-MB-435	95.00
SK-MEL-2	97.26
SK-MEL-28	112.75
SK-MEL-5	93.09
UACC-257	104.63
UACC-62	88.11
Ovarian Cancer	
IGROV1	90.21
OVCAR-3	103.23
OVCAR-4	93.65
OVCAR-5	99.84
OVCAR-8	101.61
NCI/ADR-RES	102.11
SK-OV-3	101.32
Renal Cancer	
786-0	95.99
A498	94.47
ACHN	100.08
CAKI-1	91.00
RXF 393	95.77
SN12C	99.43
TK-10	102.03
UO-31	77.22
Prostate Cancer	
PC-3	92.67
DU-145	107.74
Breast Cancer	
MCF7	98.06
MDA-MB-231/ATCC	105.41
HS 578T	93.91
T-47D	92.14
MDA-MB-468	99.87
Mean	97.30
Delta	20.08
Range	35.53

Mean Growth Percent - Growth Percent

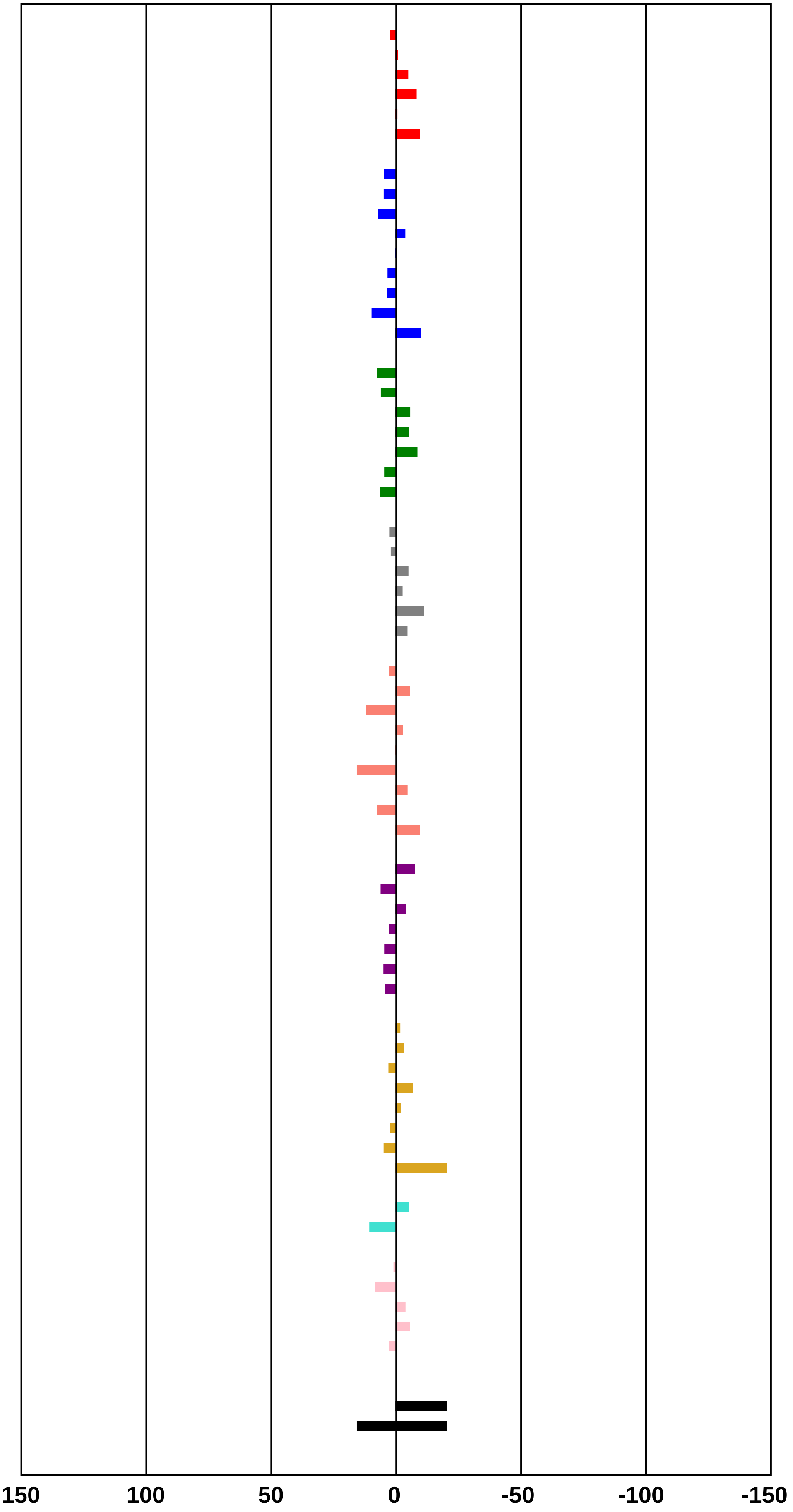

Developmental Therapeutics Program
One Dose Mean Graph 5d

NSC: D-808671 / 1	Conc: 1.00E-5 Molar	Test Date: Oct 01, 2018
Experiment ID: 1810OS18		Report Date: Oct 28, 2018

Panel/Cell Line	Growth Percent
**Leukemia**	
CCRF-CEM	102.17
HL-60(TB)	88.51
K-562	94.64
MOLT-4	81.26
RPMI-8226	107.46
SR	85.23
**Non-Small Cell Lung Cancer**	
A549/ATCC	95.42
EKVX	108.09
HOP-62	114.34
HOP-92	109.36
NCI-H226	105.47
NCI-H23	94.93
NCI-H322M	108.41
NCI-H460	108.03
NCI-H522	89.92
**Colon Cancer**	
COLO 205	117.47
HCC-2998	100.29
HCT-116	99.33
HCT-15	90.64
HT29	103.17
KM12	102.56
SW-620	113.05
**CNS Cancer**	
SF-268	105.63
SF-295	105.97
SF-539	101.82
SNB-19	103.38
SNB-75	92.63
U251	98.99
**Melanoma**	
LOX IMVI	98.96
MALME-3M	99.04
M14	110.24
MDA-MB-435	92.89
SK-MEL-2	102.16
SK-MEL-28	118.17
SK-MEL-5	96.95
UACC-257	104.89
UACC-62	99.44
**Ovarian Cancer**	
IGROV1	99.18
OVCAR-3	111.25
OVCAR-4	90.61
OVCAR-5	103.37
OVCAR-8	98.30
NCI/ADR-RES	99.33
SK-OV-3	100.00
**Renal Cancer**	
786-0	100.22
A498	103.12
ACHN	108.14
CAKI-1	93.94
RXF 393	116.41
SN12C	94.76
TK-10	104.36
UO-31	87.03
**Prostate Cancer**	
PC-3	96.50
DU-145	104.56
**Breast Cancer**	
MCF7	103.74
MDA-MB-231/ATCC	104.11
HS 578T	117.17
BT-549	105.04
T-47D	101.03
MDA-MB-468	110.54
Mean	101.73
Delta	20.47
Range	36.91

Mean Growth Percent - Growth Percent

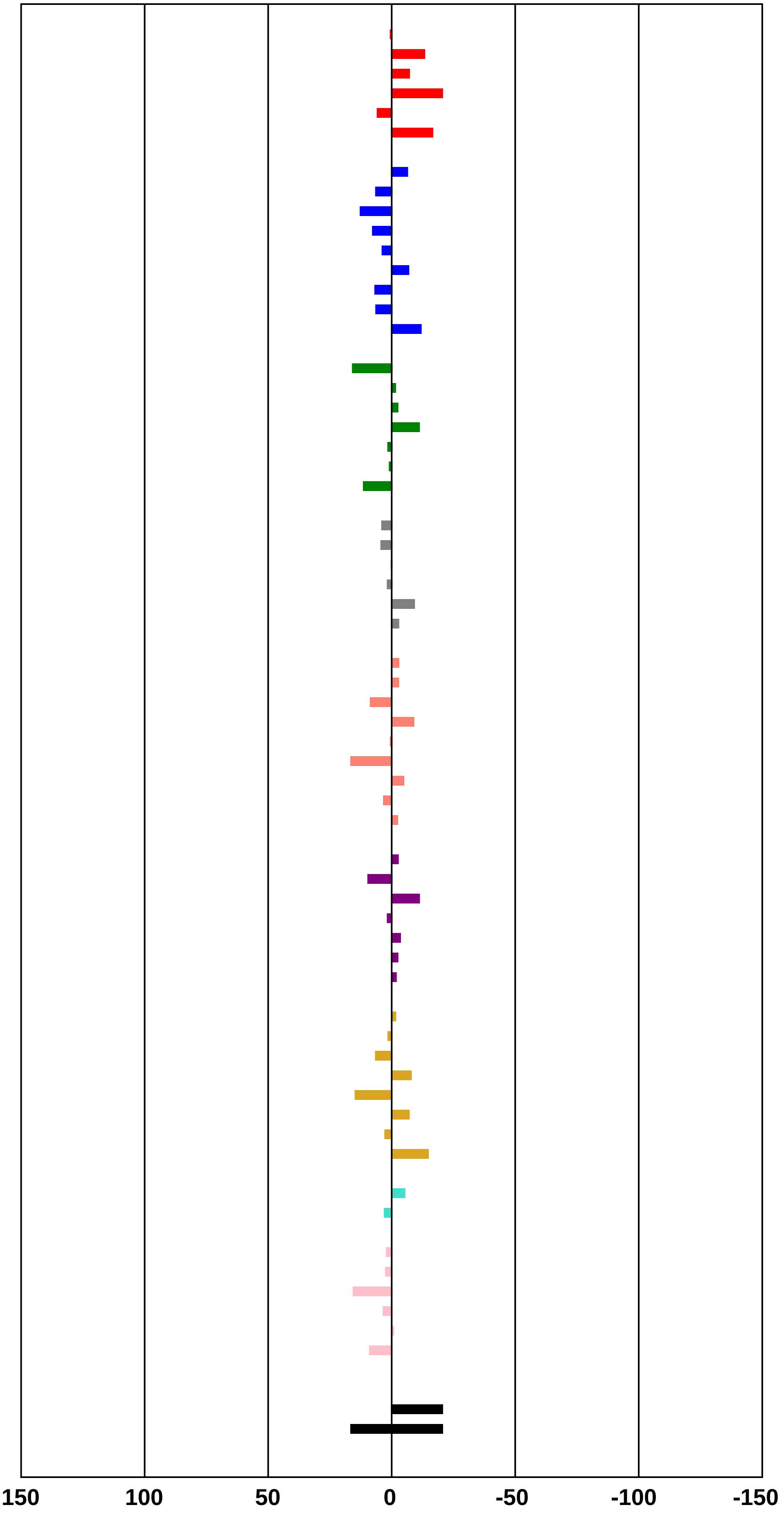

Developmental Therapeutics Program

One Dose Mean Graph 5e

**NSC:** D-808743 / 1	**Conc:** 1.00E-5 Molar		**Test Date:** Oct 01, 2018
**Experiment ID:** 1810OS18			**Report Date:** Oct 28, 2018

Panel/Cell Line	Growth Percent
**Leukemia**	
CCRF-CEM	106.12
HL-60(TB)	99.17
K-562	100.73
MOLT-4	103.53
RPMI-8226	107.02
SR	88.45
**Non-Small Cell Lung Cancer**	
A549/ATCC	102.53
EKVX	104.69
HOP-62	103.95
HOP-92	109.45
NCI-H226	104.60
NCI-H23	99.64
NCI-H322M	110.38
NCI-H460	105.47
NCI-H522	90.46
**Colon Cancer**	
COLO 205	109.65
HCC-2998	106.38
HCT-116	105.42
HCT-15	104.34
HT29	100.49
KM12	105.92
SW-620	101.09
**CNS Cancer**	
SF-268	113.00
SF-295	112.40
SF-539	96.76
SNB-19	100.38
SNB-75	95.55
U251	99.42
**Melanoma**	
LOX IMVI	99.10
MALME-3M	108.52
M14	106.53
MDA-MB-435	97.53
SK-MEL-2	102.28
SK-MEL-28	105.89
SK-MEL-5	99.99
UACC-257	101.78
UACC-62	92.67
**Ovarian Cancer**	
IGROV1	94.45
OVCAR-3	111.32
OVCAR-4	110.63
OVCAR-5	108.27
OVCAR-8	99.38
NCI/ADR-RES	106.50
SK-OV-3	97.14
**Renal Cancer**	
786-0	105.19
A498	101.71
ACHN	101.75
CAKI-1	98.14
RXF 393	109.71
SN12C	97.08
TK-10	106.95
UO-31	86.39
**Prostate Cancer**	
PC-3	98.91
DU-145	113.49
**Breast Cancer**	
MCF7	98.50
MDA-MB-231/ATCC	110.05
HS 578T	102.39
T-47D	101.02
MDA-MB-468	107.89
Mean	102.85
Delta	16.46
Range	27.10

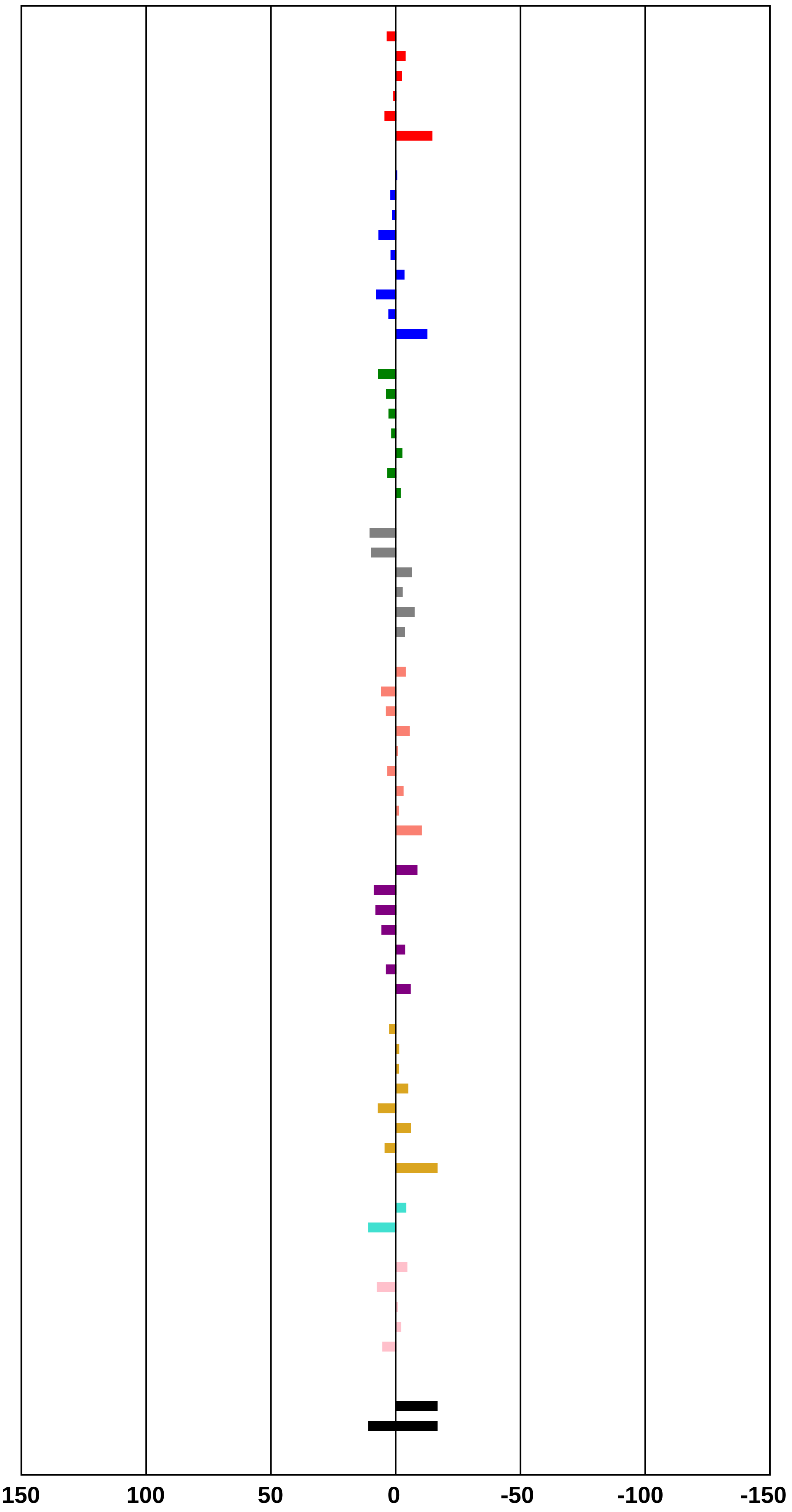

Developmental Therapeutics Program One Dose Mean Graph 5f	NSC: D-808745 / 1	Conc: 1.00E-5 Molar	Test Date: Oct 01, 2018
	Experiment ID: 1810OS18		Report Date: Oct 28, 2018

Panel/Cell Line	Growth Percent
**Leukemia**	
CCRF-CEM	107.30
HL-60(TB)	102.72
K-562	116.17
MOLT-4	114.40
RPMI-8226	109.25
SR	95.41
**Non-Small Cell Lung Cancer**	
A549/ATCC	94.86
EKVX	97.29
HOP-62	116.71
HOP-92	114.01
NCI-H226	98.44
NCI-H23	100.86
NCI-H322M	101.58
NCI-H460	108.80
NCI-H522	88.16
**Colon Cancer**	
COLO 205	118.24
HCC-2998	110.04
HCT-116	104.30
HCT-15	99.13
HT29	106.02
KM12	108.53
SW-620	106.66
**CNS Cancer**	
SF-268	113.62
SF-295	109.75
SF-539	98.19
SNB-19	102.32
SNB-75	92.94
U251	100.22
**Melanoma**	
LOX IMVI	99.75
MALME-3M	104.48
M14	111.13
MDA-MB-435	96.95
SK-MEL-2	102.05
SK-MEL-28	108.79
SK-MEL-5	101.62
UACC-257	99.64
UACC-62	97.22
**Ovarian Cancer**	
IGROV1	91.91
OVCAR-3	113.40
OVCAR-4	103.81
OVCAR-5	105.62
OVCAR-8	100.31
NCI/ADR-RES	108.87
SK-OV-3	101.45
**Renal Cancer**	
786-0	102.91
A498	110.63
ACHN	103.48
CAKI-1	92.29
RXF 393	107.77
SN12C	94.77
TK-10	112.12
UO-31	82.55
**Prostate Cancer**	
PC-3	96.32
DU-145	111.49
**Breast Cancer**	
MCF7	87.58
MDA-MB-231/ATCC	104.56
HS 578T	105.99
T-47D	100.82
MDA-MB-468	74.85
Mean	102.90
Delta	28.05
Range	43.39

Mean Growth Percent - Growth Percent

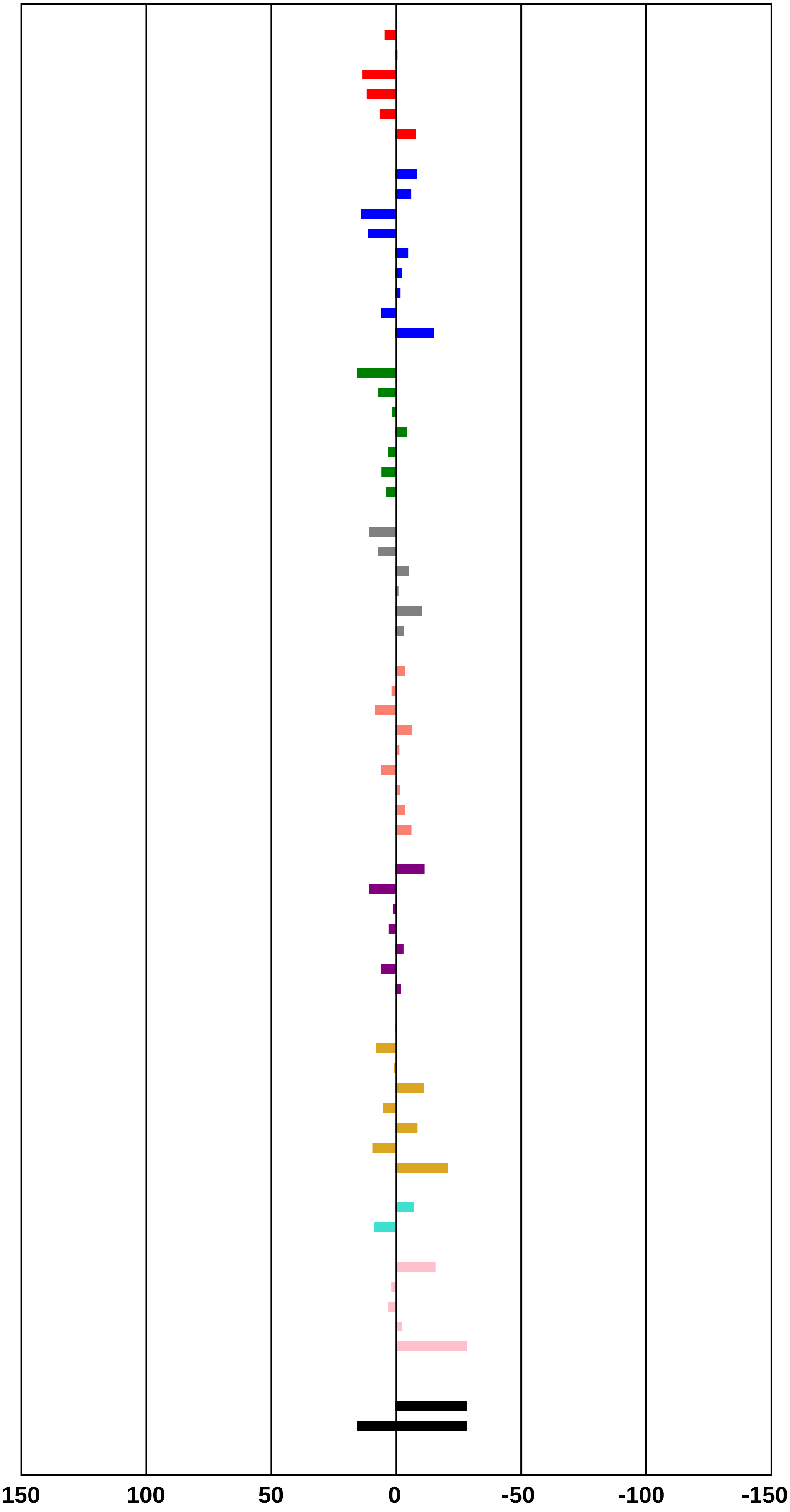

Developmental Therapeutics Program	NSC: D-808744 / 1	Conc: 1.00E-5 Molar	Test Date: Oct 01, 2018
One Dose Mean Graph 5g	Experiment ID: 1810OS18		Report Date: Oct 28, 2018

Panel/Cell Line	Growth Percent
**Leukemia**	
CCRF-CEM	108.86
HL-60(TB)	100.80
K-562	107.50
MOLT-4	106.91
RPMI-8226	106.66
SR	82.62
**Non-Small Cell Lung Cancer**	
A549/ATCC	105.62
EKVX	103.15
HOP-62	101.82
HOP-92	126.08
NCI-H226	93.34
NCI-H23	101.66
NCI-H322M	110.21
NCI-H460	106.75
NCI-H522	89.11
**Colon Cancer**	
COLO 205	109.73
HCC-2998	105.84
HCT-116	109.12
HCT-15	101.21
HT29	105.51
KM12	104.46
SW-620	102.50
**CNS Cancer**	
SF-268	103.81
SF-295	102.11
SF-539	92.56
SNB-19	100.22
SNB-75	103.02
U251	96.32
**Melanoma**	
LOX IMVI	100.35
MALME-3M	101.36
M14	113.25
MDA-MB-435	106.99
SK-MEL-2	102.01
SK-MEL-28	109.46
SK-MEL-5	99.33
UACC-257	101.12
UACC-62	94.33
**Ovarian Cancer**	
IGROV1	91.60
OVCAR-3	107.26
OVCAR-4	104.81
OVCAR-5	103.28
OVCAR-8	97.10
NCI/ADR-RES	104.16
SK-OV-3	97.14
**Renal Cancer**	
786-0	109.47
A498	109.10
ACHN	99.84
CAKI-1	104.01
RXF 393	117.90
SN12C	97.60
TK-10	109.62
UO-31	77.87
**Prostate Cancer**	
PC-3	102.74
DU-145	117.13
**Breast Cancer**	
MCF7	106.40
MDA-MB-231/ATCC	105.08
HS 578T	94.79
T-47D	100.37
MDA-MB-468	111.44
Mean	103.13
Delta	25.26
Range	48.21

Mean Growth Percent - Growth Percent

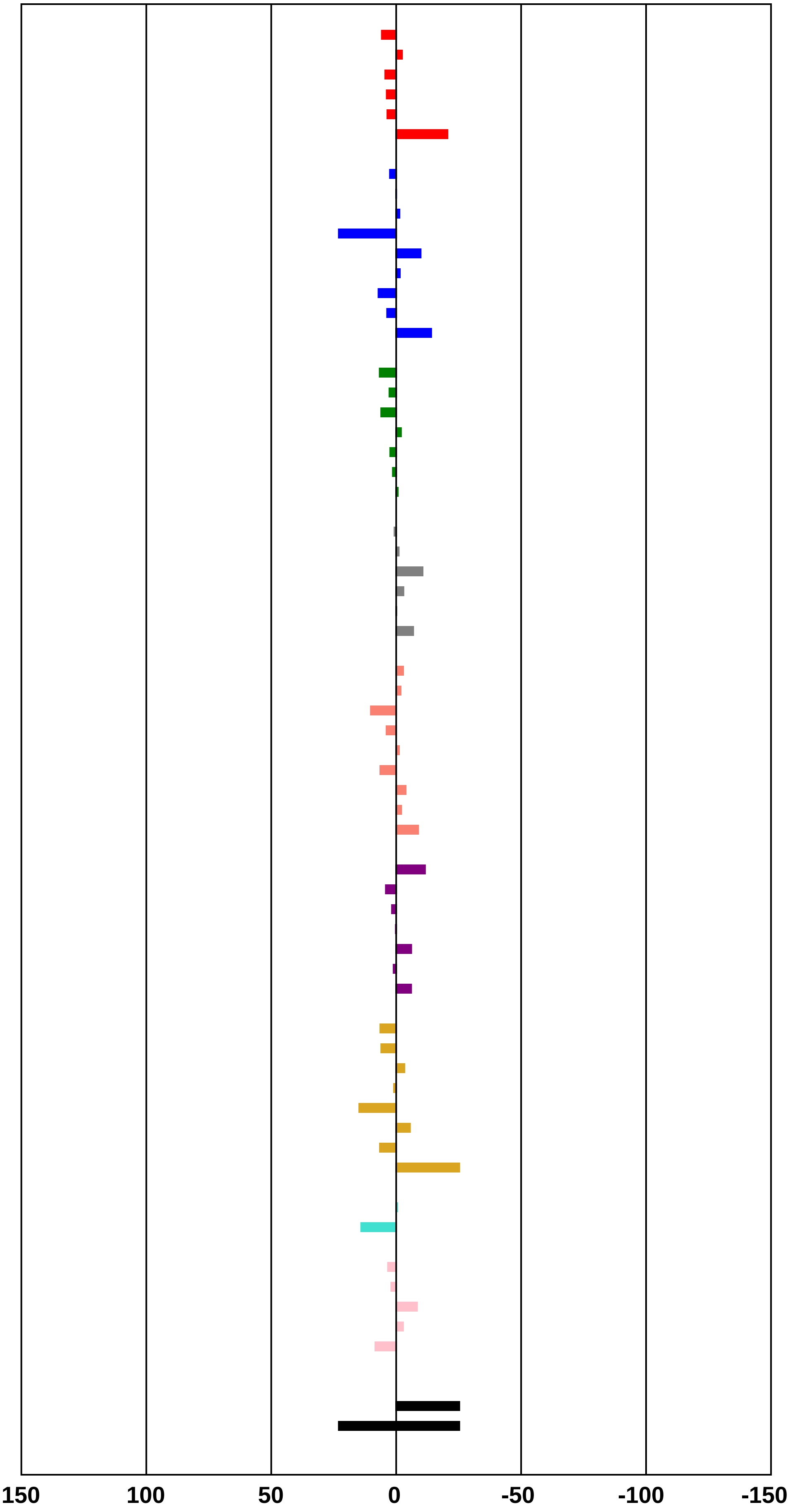

Developmental Therapeutics Program
One Dose Mean Graph

5h

NSC: D-808742 / 1	Conc: 1.00E-5 Molar	Test Date: Oct 01, 2018
Experiment ID: 1810OS18		Report Date: Oct 28, 2018

Panel/Cell Line	Growth Percent
Leukemia	
CCRF-CEM	96.86
HL-60(TB)	94.63
K-562	96.10
MOLT-4	88.92
RPMI-8226	84.73
Non-Small Cell Lung Cancer	
A549/ATCC	90.61
EKVX	97.99
HOP-62	95.18
HOP-92	70.09
NCI-H226	101.91
NCI-H23	98.88
NCI-H322M	105.85
NCI-H460	100.85
NCI-H522	85.69
Colon Cancer	
COLO 205	108.24
HCC-2998	104.49
HCT-116	89.10
HCT-15	92.87
HT29	86.35
KM12	98.41
SW-620	105.69
CNS Cancer	
SF-268	100.16
SF-295	94.20
SF-539	90.25
SNB-19	96.43
SNB-75	81.45
U251	88.92
Melanoma	
LOX IMVI	94.86
MALME-3M	93.18
M14	114.03
MDA-MB-435	99.27
SK-MEL-2	97.37
SK-MEL-28	113.87
SK-MEL-5	82.28
UACC-257	99.38
UACC-62	94.98
Ovarian Cancer	
IGROV1	81.00
OVCAR-3	107.77
OVCAR-4	86.17
OVCAR-5	100.02
OVCAR-8	95.55
NCI/ADR-RES	92.22
SK-OV-3	96.48
Renal Cancer	
786-0	98.71
A498	99.77
ACHN	90.92
CAKI-1	84.42
RXF 393	95.45
SN12C	98.58
TK-10	111.79
UO-31	65.23
Prostate Cancer	
PC-3	90.91
DU-145	99.88
Breast Cancer	
MCF7	97.10
MDA-MB-231/ATCC	97.36
HS 578T	76.48
T-47D	85.77
MDA-MB-468	98.65
Mean	94.56
Delta	29.33
Range	48.80

Mean Growth Percent - Growth Percent

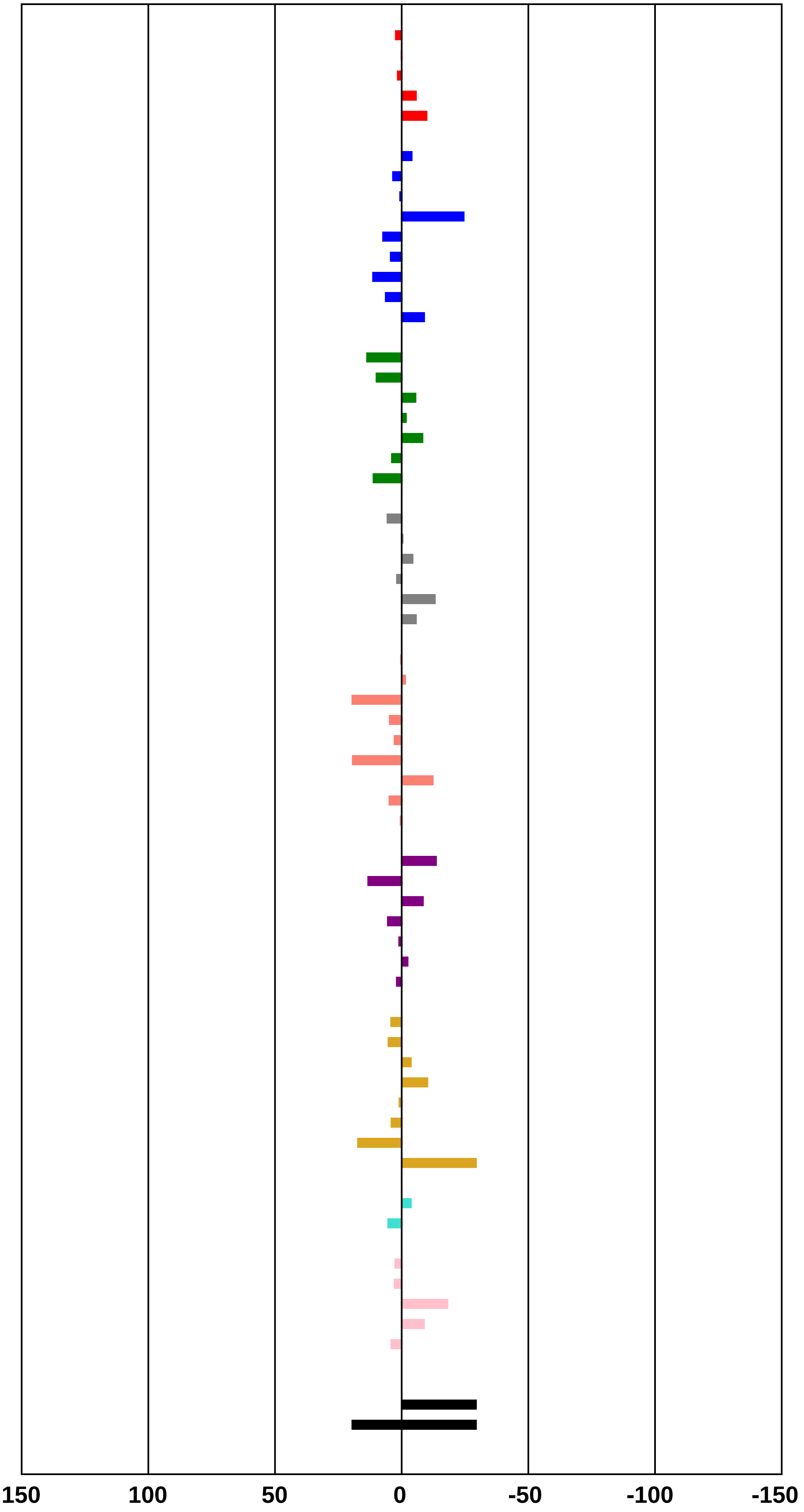

Supplement: Supplementary file 1 [file ijms-20-02484-s001.pdf]
